# Prolonged airway explant culture enables study of health, disease, and viral pathogenesis

**DOI:** 10.1126/sciadv.adp0451

**Published:** 2025-04-25

**Authors:** Rhianna E. Lee-Ferris, Kenichi Okuda, Jacob R. Galiger, Stephen A. Schworer, Troy D. Rogers, Hong Dang, Rodney Gilmore, Caitlin Edwards, Gillian Crisp, Satoko Nakano, Anne M. Cawley, Raymond J. Pickles, Samuel C. Gallant, Elisa Crisci, Lauraine Rivier, James S. Hagood, Wanda K. O’Neal, Ralph S. Baric, Barbara R. Grubb, Richard C. Boucher, Scott H. Randell

**Affiliations:** ^1^ Marsico Lung Institute/CF Center, University of North Carolina at Chapel Hill, Chapel Hill, NC, USA.; ^2^ Cell Biology and Physiology, University of North Carolina at Chapel Hill, Chapel Hill, NC, USA.; ^3^ Epidemiology, University of North Carolina at Chapel Hill, Chapel Hill, NC, USA.; ^4^ Microbiology and Immunology, University of North Carolina at Chapel Hill, Chapel Hill, NC, USA.; ^5^ College of Veterinary Medicine, Department of Population Health and Pathobiology, North Carolina State University, Raleigh, NC, USA.; ^6^ Pediatric Pulmonology and Program for Rare and Interstitial Lung Disease, University of North Carolina at Chapel Hill, Chapel Hill, NC, USA.; ^7^ Genetics, University of North Carolina at Chapel Hill, Chapel Hill, NC, USA.

## Abstract

In vitro models play a major role in studying airway physiology and disease. However, the native lung’s complex tissue architecture and nonepithelial cell lineages are not preserved in these models. Ex vivo tissue models could overcome in vitro limitations, but methods for long-term maintenance of ex vivo tissue have not been established. Here, we describe methods to culture human large airway explants, small airway explants, and precision-cut lung slices for at least 14 days. Human airway explants recapitulate genotype-specific electrophysiology; characteristic epithelial, endothelial, stromal, and immune cell populations; and model viral infection after 14 days in culture. These methods also maintain mouse, rabbit, and pig tracheal explants. Notably, intact airway tissue can be cryopreserved, thawed, and used to generate viable explants with recovery of function 14 days postthaw. These studies highlight the broad applications of airway tissue explants and their use as translational intermediates between in vitro and in vivo studies.

## Teaser

Ex vivo models capture the cellular and functional richness of respiratory tract tissues and enable study of viral pathogenesis.

## INTRODUCTION

Airway epithelia constitute an important barrier between the body and the environment, serving as the first point of contact with inhaled chemicals, particles, and pathogens. Airway barrier function is crucial for health, and barrier failure often leads to disease. Air-liquid interface (ALI) cultures have long served to model airway epithelial barrier function and physiology (*1*, *2*). In this system, airway epithelial cells form a pseudostratified epithelium containing the major cell types of in vivo airways, including basal, secretory, and ciliated cells. However, in vivo stromal architecture, submucosal glands (SMGs), and epithelial interactions with tissue-resident immune cells are not preserved. Animal models offer another strategy to study the respiratory epithelium. However, translating findings from animal models to humans is not always straightforward.

Airway explants contain diverse cell types and spatial information characteristic of the in vivo tissue and, thus, may serve as an intermediate between ALI cultures and in vivo models. Despite these advantages, explant culture methods typically require fresh tissue obtained within a few hours of resection (*3*). Methods to recover and culture human tissues after longer cold ischemic times have not been developed. Further, explants have been historically limited by short-term viability (~4 days) (*4*–*8*) and submerged culture methods, which do not mimic the in vivo air-tissue interface.

Here, we developed methods to culture explants from three major lung regions, including bronchi [large airway explants (LAEs)], bronchioles [small airway explants (SAEs)], and alveolar parenchyma [precision cut lung slices (PCLSs)]. Maintaining explants on a Gelfoam sponge enabled an ALI, circumventing the need for submerged culture. Even after long cold ischemic times (up to 40 hours), cultured airway explants remained viable and retained in vivo–like characteristics for 14 days. We assessed explant cellular composition, electrophysiology, and cryopreservation and demonstrated their utility to study viral infection.

## RESULTS

### Gelfoam culture maintains in vivo–like tissue architecture and characteristic cell types

**Fig. 1.**
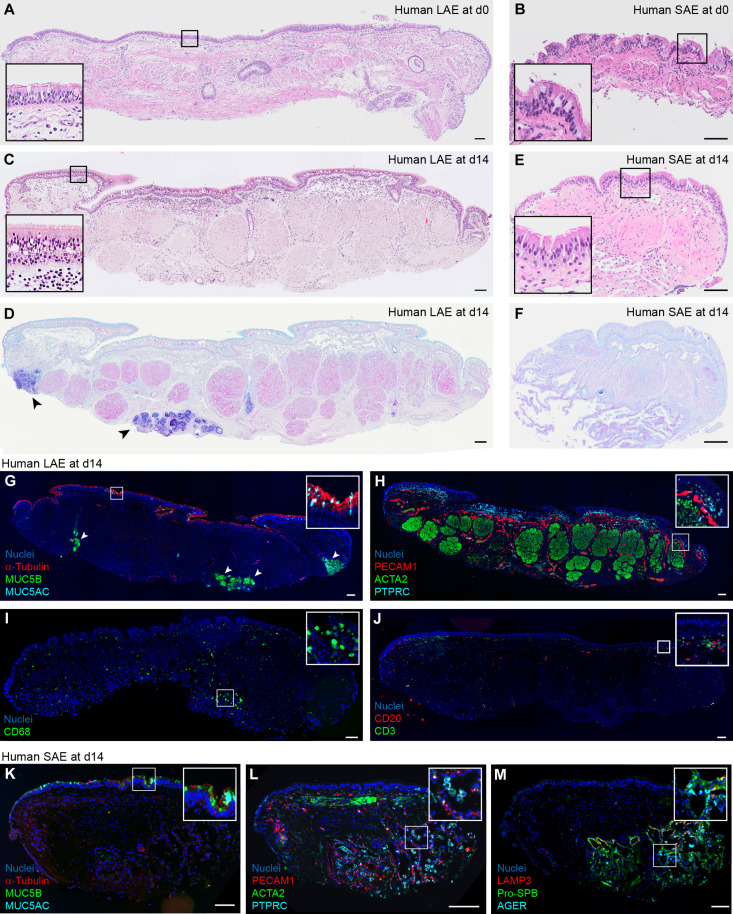
Human LAEs and SAEs exhibit native tissue architecture and characteristic epithelial, mesenchymal, and immune cell types after 14 days in culture. (**A** and **B**) Hematoxylin and eosin (H&E) staining of human LAEs (A) and SAEs (B) immediately after dissection [day 0 (d0)]. Representative of *n* = 7 and 5 donors, respectively. (**C** and **D**) H&E (C) and AB-PAS (D) staining of a human LAE explant after 14 days in culture. Arrowheads in (D) indicate SMGs. Representative of *n* = 17 donors. (**E** and **F**) H&E (E) and AB-PAS (F) of a human SAE explant after 14 days in culture. Representative of *n* = 13 donors. (**G** to **J**) IF localization of α-tubulin, MUC5B, and MUC5AC (G); PECAM1, ACTA2, and PTPRC (H); CD68 (I); and CD20 and CD3 (J) in day 14 LAE explants. Arrowheads in (G) indicate SMGs. [(G) to (J)] Representative of *n* = 3 donors. (**K** to **M**) IF localization of α-tubulin, MUC5B, and MUC5AC (K); PECAM1, ACTA2, and PTPRC (L); and LAMP3, pro-SPB, and AGER (M) in day 14 SAE explants. [(K) to (M)] Representative of *n* = 3 donors. Scale bars, 100 μm.

The epithelium of human LAEs and SAEs was examined immediately after dissection and after 14 days of culture on Gelfoam at an ALI (see the Materials and Methods and Fig. 1, A to F). Much similar to the freshly explanted tissue (Fig. 1, A and B), the day 14 LAE and SAE epithelium was pseudostratified and well ciliated, with SMGs containing alcian blue–periodic acid–Schiff (AB-PAS)–positive cells and secretions visible in LAEs (Fig. 1, C to F). By immunofluorescence (IF), abundant acetylated α-tubulin^+^ ciliated cells and sparse mucin 5AC (MUC5AC)^+^ goblet cells were observed in the surface epithelium, along with robust MUC5B staining in the SMGs of day 14 LAEs (Fig. 1G). Bundles of actin alpha 2 smooth muscle (ACTA2)^+^ smooth muscle cells, platelet and endothelial cell adhesion molecule 1 (PECAM1)^+^ endothelial cells (ECs), and protein tyrosine phosphatase receptor type C (PTPRC)^+^ leukocytes were present below the epithelium (Fig. 1H). Macrophages, positive for CD68, T cells (CD3^+^), and B cells (CD20^+^) were also present in day 14 LAE tissues (Fig. 1, I and J). SAEs at day 14 also expressed α-tubulin, MUC5B, MUC5AC, ACTA2, PECAM1, and PTPRC (Fig. 1, K and L) and retained viable alveolar tissue on the Gelfoam-facing side of the explant, as indicated by IF for the alveolar type 2 (AT2) cell markers, lysosomal-associated membrane protein 3 (LAMP3) and prosurfactant protein B (pro-SPB), and the AT1 cell marker, advanced glycosylation end-product specific receptor (AGER) (Fig. 1M).

Maintenance of characteristic airway epithelial cells was confirmed by RNA in situ hybridization. Abundant *Forkhead box J1* (*FOXJ1*) signal, marking ciliated cells, and *Mucin 5B* (*MUC5B*) signal marking secretory cells in the surface epithelium of LAEs and SAEs as well as in the gland ducts and acini in LAE tissues were observed (fig. S1, A to B and E to F). The secretory cell marker, *Secretoglobin Family 1A Member 1* (*SCGB1A1*), was also present in LAE and SAE tissues, with higher abundance in SAEs as previously reported (*9*) (fig. S1, C and G). *Keratin 5* (*KRT5*), a marker of airway basal cells, was present in the surface epithelium and gland ducts of LAEs (fig. S1D). We also detected expression of *Surfactant Protein B* (*SFTPB*), a marker of distal airway secretory cells and AT2 cells, in both the airway surface epithelium and alveolar parenchyma of SAE tissues (fig. S1H). Overall, LAEs and SAEs appeared to preserve characteristic epithelial, endothelial, and immune cell populations for at least 14 days in culture.

### Human PCLSs cultured on Gelfoam preserve native tissue architecture and characteristic cell types

**Fig. 2.**
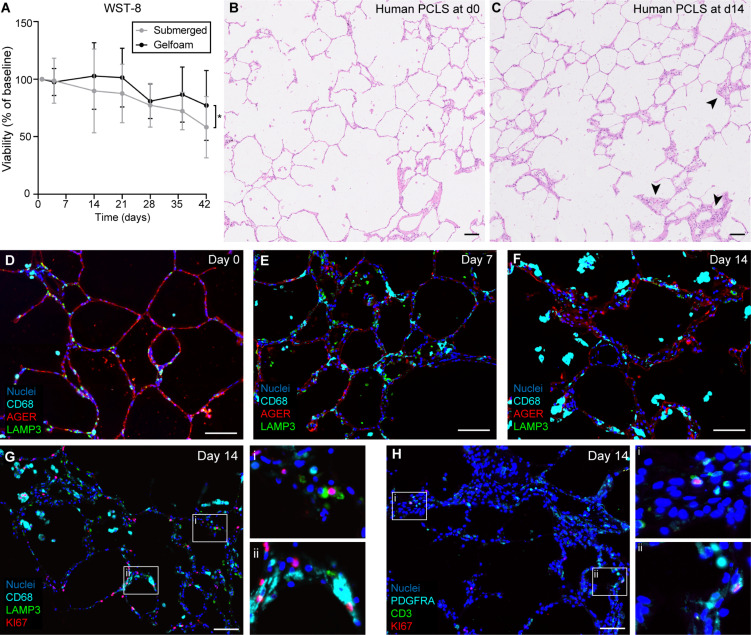
Human PCLSs preserve diverse cell lineages for 14 days of culture on Gelfoam. (**A**) WST-8 viability assessment of human PCLSs cultured at ALI on Gelfoam or under traditional submerged methods. *n* = 3 donors; three replicates per donor. Gelfoam-cultured PCLSs had significantly greater viability using a linear mixed-effects model with the donor as a random effects factor. *P < 0.05. (**B** and **C**) H&E histology of day 0 (B) and day 14 (C) human PCLSs. [(B) and (C)] Representative of *n* = 8 donors. (**D** to **F**) IF localization of CD68, AGER, and LAMP3 in human PCLSs at day 0 (D), day 7 (E), and day 14 (F). [(D) to (F)] Representative of *n* = 3 donors. (**G** and **H**) IF localization of KI67 with CD68 and LAMP3 (G) and with CD3 and platelet-derived growth factor receptor A (PDGFRA) (H) in day 14 human PCLSs. [(G) and (H)] Representative of *n* = 3 donors. Scale bars, 100 μm.

In vitro model systems to study the alveolar epithelium over extended time intervals are less established compared to the upper airways. PCLSs preserve the alveolar epithelium within the context of its native, complex tissue architecture and have been widely adopted to study airway constriction, toxicology, nanotechnology, and viral pathogenesis (*10*–*14*). However, most PCLS studies are limited to short-term culture (≤72 hours) (*12*, *15*, *16*). We hypothesized that culturing PCLSs on Gelfoam at an ALI would extend tissue viability while maintaining in vivo tissue functional properties. To test this hypothesis, we measured the viability of PCLSs cultured on Gelfoam and under conventional submerged culture conditions using a WST-8 cell counting kit (Fig. 2A). Submerged PCLS viability began decreasing after 4 days under standard culture conditions, whereas the viability of PCLSs on Gelfoam remained stable for 21 days before decreasing. Hematoxylin and eosin (H&E) staining of day 0 and day 14 Gelfoam-cultured PCLSs revealed preservation of intact alveolar structures (Fig. 2, B and C), but in some regions of day 14 PCLSs, larger patches of cells were seen in the alveolar interstitium (Fig. 2C, arrowheads), reflecting increased numbers of unknown cell types.

To identify this population, we assessed the cell type composition of PCLSs cultured for 0, 7, and 14 days by IF (Fig. 2, D to F). At all three time points, CD68^+^ macrophages, AGER^+^ AT1 cells, and LAMP3^+^ AT2 cells were observed. RNA in situ hybridization further confirmed *CD68*, *AGER*, and *SFTPB* expression in day 14 PCLSs (fig. S1, I to K). Next, we performed IF localization for the proliferation marker KI67 in day 0 and day 14 tissues. KI67 signal was nearly absent from day 0 tissues (fig. S1, L and M) but colocalized with a subset of CD68^+^ macrophages (Fig. 2G), LAMP3^+^ AT2 cells (Fig. 2G), CD3^+^ T cells (Fig. 2H), and platelet-derived growth factor receptor A (PDGFRA)^+^ fibroblasts (Fig. 2H) in day 14 PCLSs. From this, we concluded that the increased cellularity in day 14 PCLSs resulted from cell proliferation across multiple cell lineages. Collectively, these data indicate that ALI culture of PCLSs on Gelfoam was superior to conventional submerged culture methods, and this method was used for all remaining experiments.

### LAEs and SAEs exhibit in vivo–like electrophysiology after 14 days in culture

**Fig. 3.**
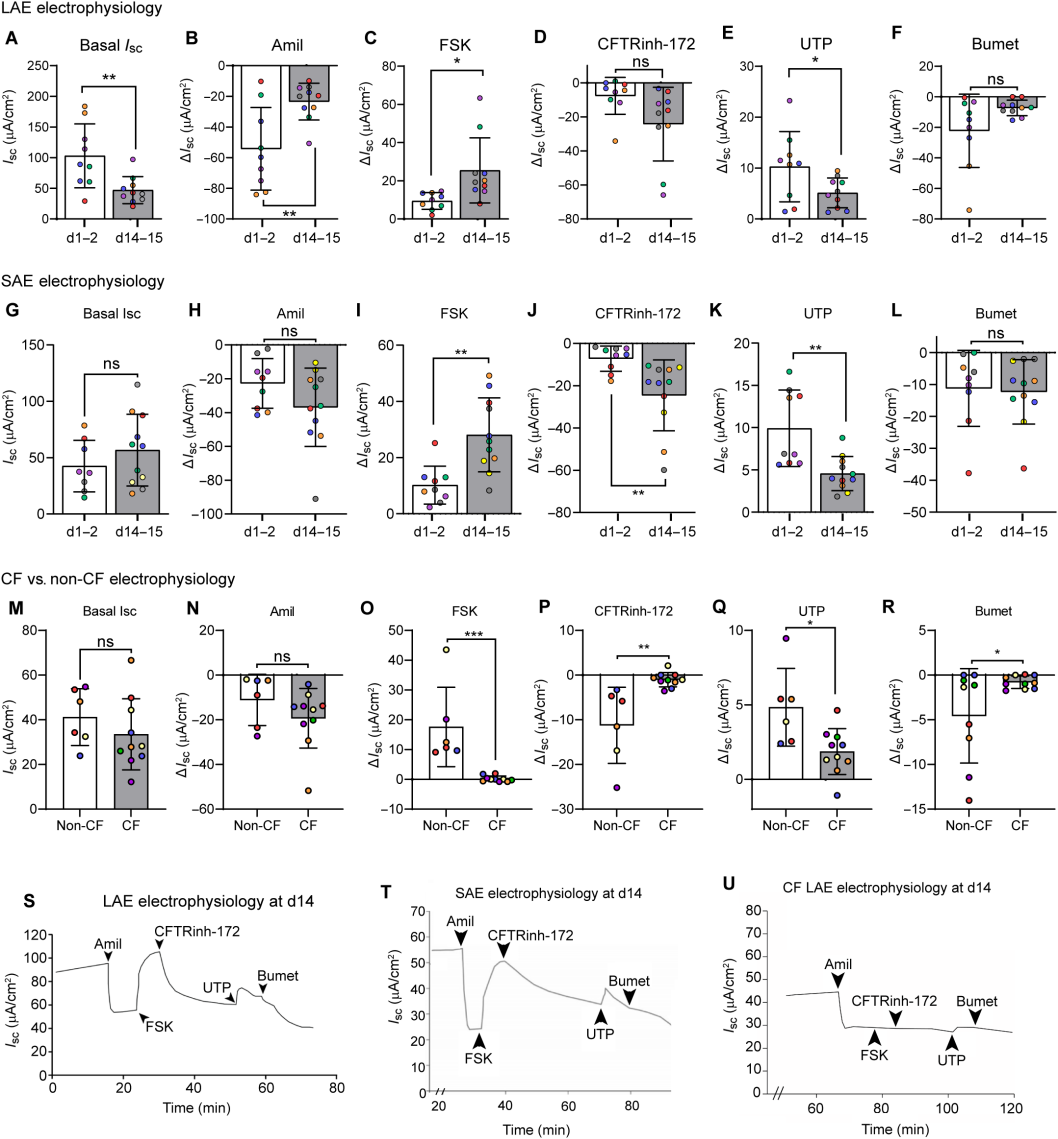
Human airway explants retain genotype-specific electrophysiology for 14 days in culture. (**A** to **F**) Electrophysiology of human LAE explants at days 1 and 2 and days 14 and 15. (A) Basal short circuit current (*I*_sc_) and Δ*I*_sc_ in response to (B) Amil, (C) FSK, (D) CFTRinh-172, (E) uridine 5′-triphosphate (UTP), and (F) bumetanide (Bumet). *n* = 5 to 6 donors (represented by different colored dots); one to two replicates per donor. (**G** to **L**) Electrophysiology of human SAE explants at days 1 and 2 and days 14 and 15. (G) Basal *I*_sc_ and Δ*I*_sc_ in response to (H) Amil, (I) FSK, (J) CFTRinh-172, (K) UTP, and (L) bumetanide. *n* = 6 donors (represented by different colored dots); one to two replicates per donor. (**M** to **R**) Electrophysiology of human CF versus non-CF LAE explants at day 14. (M) Basal *I*_sc_ and Δ*I*_sc_ in response to (N) Amil, (O) FSK, (P) CFTRinh-172, (Q) UTP, and (R) bumetanide. *n* = 5 non-CF and 6 CF donors (represented by different colored dots); one to two replicates per donor. (**S** to **U**) Representative Ussing tracing of day 14 human LAE explant (S), SAE explant (T), and CF LAE explant (U). [(A) to (R)] Unpaired *t* test. *P < 0.05 and **P < 0.01. ns, nonsignificant.

Explant viability and function were investigated by assessing electrophysiology in Ussing chambers. Electrophysiology was not measurable immediately after dissection, likely due to the long cold ischemic times of the studied tissues (up to 40 hours between surgical resection and placement on Gelfoam). However, LAE and SAE electrophysiology recovered after 1 to 2 days or 14 to 15 days in culture (Fig. 3, A to L and S to T). When equilibrated in bilateral Krebs-Ringer Bicarbonate (KBR) solution, the mean basal short circuit current (*I*_sc_) of LAEs and SAEs were 112.1 and 42.4 μA/cm^2^, respectively, at days 1 and 2 and 49.7 and 56.7 μA/cm^2^, respectively, at days 14 and 15 (Fig. 3, A and G). The day 14 and day 15 measurements were notably similar to measurements from previous studies of freshly excised bronchi and bronchioles that exhibited basal *I*_sc_ of 51 and 55 μA/cm^2^, respectively (*8*, *17*). By contrast, the twofold greater basal *I*_sc_ and amiloride (Amil) response along with the muted forskolin (FSK) response at days 1 and 2 likely reflects ongoing recovery from cold ischemia following surgical resection.

In day 14 and day 15 explants, robust responses to Amil indicated epithelial sodium channel (ENaC) activity, and responses to FSK and cystic fibrosis (CF) transmembrane regulator (CFTR) inhibitor–172 (CFTRinh-172) indicated CFTR activity (Fig. 3, B to D and H to J), again aligning with previous work (*8*, *17*). Last, we compared CF and non-CF LAEs after 14 days of culture and observed no CFTR activity in CF LAEs as expected, indicating that the disease phenotype of reduced CFTR function was preserved by explant culture (Fig. 3, M to R, S, and U). Collectively, these data suggest that Gelfoam culture methods enable culture of viable airway explants that model in vivo–like electrophysiology after 14 days in culture.

### Single-cell RNA sequencing reveals diverse cell lineages preserved in the LAE, SAE, and PCLS models

**Fig. 4.**
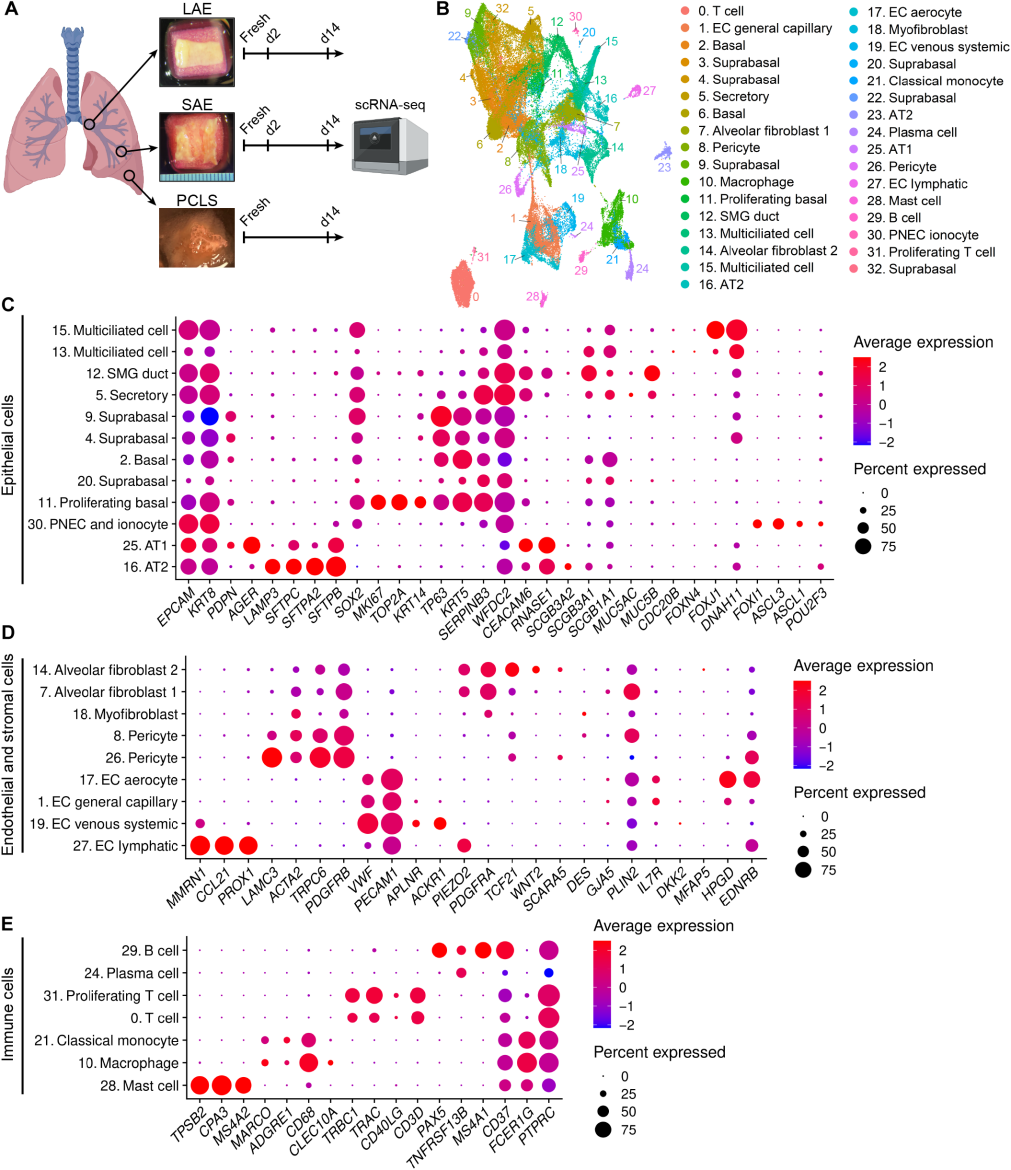
scRNA-seq of LAE, SAE, and PCLS models over time. (**A**) Schematic of scRNA-seq on LAEs, SAEs, and PCLSs at day 0 (fresh), day 2, and day 14. (**B**) Uniform Manifold Approximation and Projection indicating 33 identified cell clusters. (**C** to **E**) Dot plots showing the top differentially expressed genes in epithelial (C), endothelial and stromal (D), and immune cell (E) clusters.

To query cell population dynamics in the Gelfoam model, we performed single-cell RNA sequencing (scRNA-seq) on LAEs, SAEs, and PCLSs from *n* = 3 donors. After 0, 2, or 14 days in culture, samples were preserved in CryoStor commercial freezing medium and processed as one batch per donor before combining and integrating the data (Fig. 4A and fig. S2). Using the quality control metrics defined in Materials and Methods, we obtained a total of 85,305 cells combining all three regions and time points. A graph-based clustering approach using Seurat v4 produced 33 distinct clusters (Fig. 4B). We then referenced the Human Lung Cell Atlas dataset (*18*) to assign prediction scores for 61 distinct cell signatures. Clusters 3, 6, 22, 23, and 32 contained cells from a single donor only and were not considered representative of the model as a whole (fig. S2F).

Multiple epithelial cell clusters, including basal cells, suprabasal cells, secretory cells, SMG duct cells, multiciliated cells, AT1s, AT2s, pulmonary neuroendocrine cells (PNECs), and ionocytes were identified in our dataset based on characteristic marker expression (Fig. 4C). EC clusters included EC capillary, venous systemic ECs, lymphatic ECs, and the recently described EC aerocytes (*19*) (Fig. 4D). Stromal cell clusters included fibroblasts, myofibroblasts, pericytes, alveolar fibroblast 1 (AF1), and AF2 (Fig. 4D). Last, immune cell clusters included macrophages, T cells (CD4^+^ and CD8^+^), B cells, mast cells, plasma cells, and classical monocytes (Fig. 4E).

**Fig. 5.**
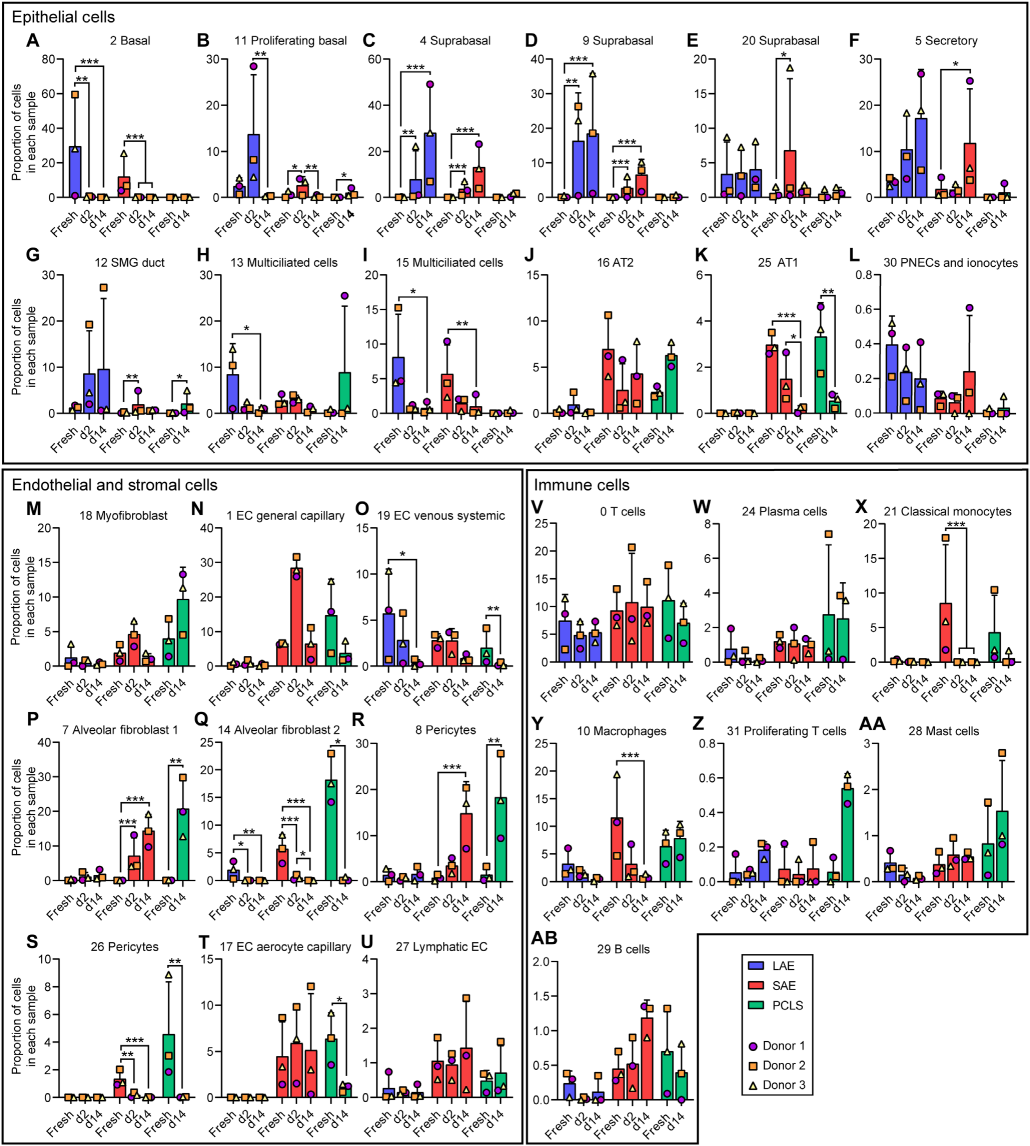
Cell population dynamics over time in the LAE, SAE, and PCLS model. Proportion of (**A**) cluster 2, basal cells; (**B**) cluster 11, proliferating basal cells; (**C**) cluster 4, suprabasal cells; (**D**) cluster 9, suprabasal cells; (**E**) cluster 20, suprabasal cells; (**F**) cluster 5, secretory cells; (**G**) cluster 12, SMG duct cells; (**H**) cluster 13, multiciliated cells; (**I**) cluster 15, multiciliated cells; (**J**) cluster 16, AT2 cells; (**K**) cluster 25, AT1 cells; (**L**) cluster 30, PNECs and ionocytes; (**M**) cluster 18, myofibroblasts; (**N**) cluster 1, EC general capillary cells; (**O**) cluster 19, EC venous systemic cells; (**P**) cluster 7, AF1; (**Q**) cluster 14, AF2; (**R**) cluster 8, pericytes; (**S**) cluster 26, pericytes; (**T**) cluster 17, EC aerocyte capillary cells; (**U**) cluster 27, lymphatic ECs; (**V**) cluster 0, T cells; (**W**) cluster 24, plasma cells; (**X**) cluster 21, classical monocytes; (**Y**) cluster 10, macrophages; (**Z**) cluster 31, proliferating T cells; (**AA**) cluster 28, mast cells; and (**AB**) cluster 29, B cells in each scRNA-seq sample. [(A) to (AB)] *n* = 3 donors; one replicate per donor. Statistical testing was performed on a sample-wise cell count matrix with a generalized linear model (GLM) fit and negative binomial (NB) distribution, using a quasi-likelihood (QL) *F* test. *False discovery rate (FDR) < 0.05, **FDR < 0.01, and ***FDR < 0.001. Unmarked comparisons are nonsignificant. See table S1 for a full summary of statistical testing.

Our data identified five distinct populations of basal or suprabasal cells (Fig. 5, A to E). These clusters were generally more abundant in LAE than SAE explants and virtually absent from PCLSs, reflecting the small numbers of conducting airways in PCLSs. These clusters expressed the basal cell markers *KRT5* and *KRT15*, as expected (fig. S3, A to B). Cluster 2 (basal) decreased over time (Fig. 5A), possibly representing a homeostatic cell state that is not preserved by explant culture methods. By contrast, cluster 11 (proliferating basal) transiently increased at day 2 but decreased again by day 14 (Fig. 5B) and expressed high levels of the proliferation markers, *MKI67* and *TOP2A* (fig. S3, C to D). This pattern was also observed by IF localization of KI67 with abundant signal at day 2 and no signal at day 15 (fig. S3, E to F). Clusters 4 and 9 (suprabasal) increased over time (Fig. 5, C to D), and expressed relatively high levels of the hypoxia markers, *EGLN3* and *P4HA1* (*20*) (fig. S3, G to H). This may indicate an epithelial response to apical hypoxia over time, potentially explained by the visible accumulation of apical mucus (fig. S3I). Cluster 20 (suprabasal) cell abundance was relatively constant in LAEs but transiently increased at day 2 in one donor in the SAE model (Fig. 5E).

The proportion of secretory cells (cluster 5) in LAE and SAE models as well as SMG duct cells (cluster 12) in the LAE model increased over time (Fig. 5, F to G), whereas the proportion of multiciliated cells (clusters 13 and 15) decreased over time in both models (Fig. 5, H to I). Our previous H&E and IF staining for α-tubulin (Fig. 1, C, E, G, and K) demonstrated a well-ciliated epithelium at day 14. Thus, we hypothesized that the proportional decrease in multiciliated cells was due to an increase in other cell types rather than ciliated cell loss over time. Expression of the ciliated cell genes *DNAH11* and *FOXJ1* remained constant in both clusters 13 and 15 (fig. S3, J to K) and quantitation of α-tubulin signal confirmed that ciliated cells are retained over 14 days in culture (fig. S3, L to Q). AT2 cells (cluster 16) increased in proportion in the PCLS model, whereas AT1 cells (cluster 25) decreased over time in both PCLS and SAE models (Fig. 5, J to K). PNECs and ionocytes (cluster 30) were low abundance and remained relatively constant over time (Fig. 5L).

With respect to endothelial and stromal cell populations, PCLSs exhibited an increase in myofibroblasts (cluster 18; Fig. 5M). General capillary ECs (cluster 1) transiently increased in SAE models at day 2 and decreased in PCLSs over time (Fig. 5N), whereas venous systemic ECs (cluster 19) decreased over time in all three models (Fig. 5O). The proportion of AF1 (cluster 7) and a subset of pericytes (cluster 8) increased over time, whereas AF2 (cluster 14) and a different subset of pericytes (cluster 26) decreased over time in SAEs and PCLSs (Fig. 5, P to S). Aerocyte capillary ECs (cluster 17) were absent in the LAE model and decreased over time in the PCLSs (Fig. 5T), whereas lymphatic ECs were relatively stable in all models (Fig. 5U).

With regard to immune cell dynamics, the proportion of T cells (clusters 0) and plasma cells (cluster 24) were constant over time across all models (Fig. 5, V and W), while classical monocytes (cluster 21) were only present in fresh tissues (Fig. 5X). The proportion of macrophages (cluster 10) decreased over time in the LAE and SAE models but was maintained in the PCLS model (Fig. 5Y), consistent with replenishment by the proliferating macrophages seen in Fig. 2G. Proliferating T cells (cluster 31) also increased in the PCLS model (Fig. 5Z), again aligning with IF staining (Fig. 2H). B cells and mast cells were present in low numbers, and the proportion remained relatively stable over time (Fig. 5, AA to AB).

Beyond analyzing cell type proportions, we also assessed shifts in the overall transcriptome over time in the major cellular compartments. Subset clusters of the major epithelial cell types were grouped as follows: basal cells (clusters 2, 4, 9, 11, and 20), ciliated cells (clusters 13 and 15), secretory cells (cluster 5), SMG duct (cluster 12), AT2s (cluster 16), AT1s (cluster 25), and rare cells (cluster 30). Endothelial and stromal cell clusters were grouped into fibroblasts (clusters 7, 14, and 18), ECs (clusters 1, 17, 19, and 27), and pericytes (clusters 8 and 26). Immune cell clusters were categorized as T cells (clusters 0 and 31), B cells (clusters 24, and 29), mast cells (cluster 28), and macrophages (clusters 10 and 21). Differentially expressed gene analyses identified 48 genes that were significantly differentially expressed between day 0 (fresh) and day 14 samples in more than 70% of cell types.

Among these, several heat shock protein (HSP) genes (*HSPA1A*, *HSPA1B*, *HSPH1*, *DNAJB*1, and *DNAJB6*) were down-regulated in day 14 cultures (fig. S4). This shift likely reflects an acute cellular stress at day 0 associated with cold ischemia and tissue dissection with an eventual adaptation to the ex vivo culture environment reducing cellular stress as the cells adjust to Gelfoam culture. Reduced form of nicotinamide adenine dinucleotide (oxidized form) (NADH) dehydrogenase subunits (*MT-ND1*, *MT-ND2*, *MT-ND3*, and *MT-ND4*) were also commonly down-regulated in day 14 cultures, whereas genes encoding glycolytic enzymes (*ENO1*, *PKM*, *GAPDH*, *LDHA*, and *TPI1*) were commonly up-regulated in day 14 cultures (fig. S4). Together, this points toward robust mitochondrial oxidative phosphorylation activity in fresh tissue with a metabolic shift toward glycolysis in day 14 cultures, potentially reflecting changes in oxygen availability under ex vivo culture conditions. Although these genes were consistently up- or down-regulated across cell types, the magnitude of regulation was largely cell type specific. For instance, down-regulation of NADH dehydrogenase subunits genes and up-regulation of glycolytic enzyme genes were most pronounced in endothelial and stromal cells compared to immune or epithelial compartments. This may indicate that stromal cells rely more heavily on systemic oxygen supply, which is absent under Gelfoam culture conditions, whereas epithelial cells are known to obtain oxygen from the apical airway lumen side (*20*–*22*). Overall, airway explant and PCLS cultures preserve diverse cell lineages over time in culture with shifts in cell proportions and metabolic and stress-related adaptations to culture conditions.

### Explant culture methods extend to animal airway tissue

**Fig. 6.**
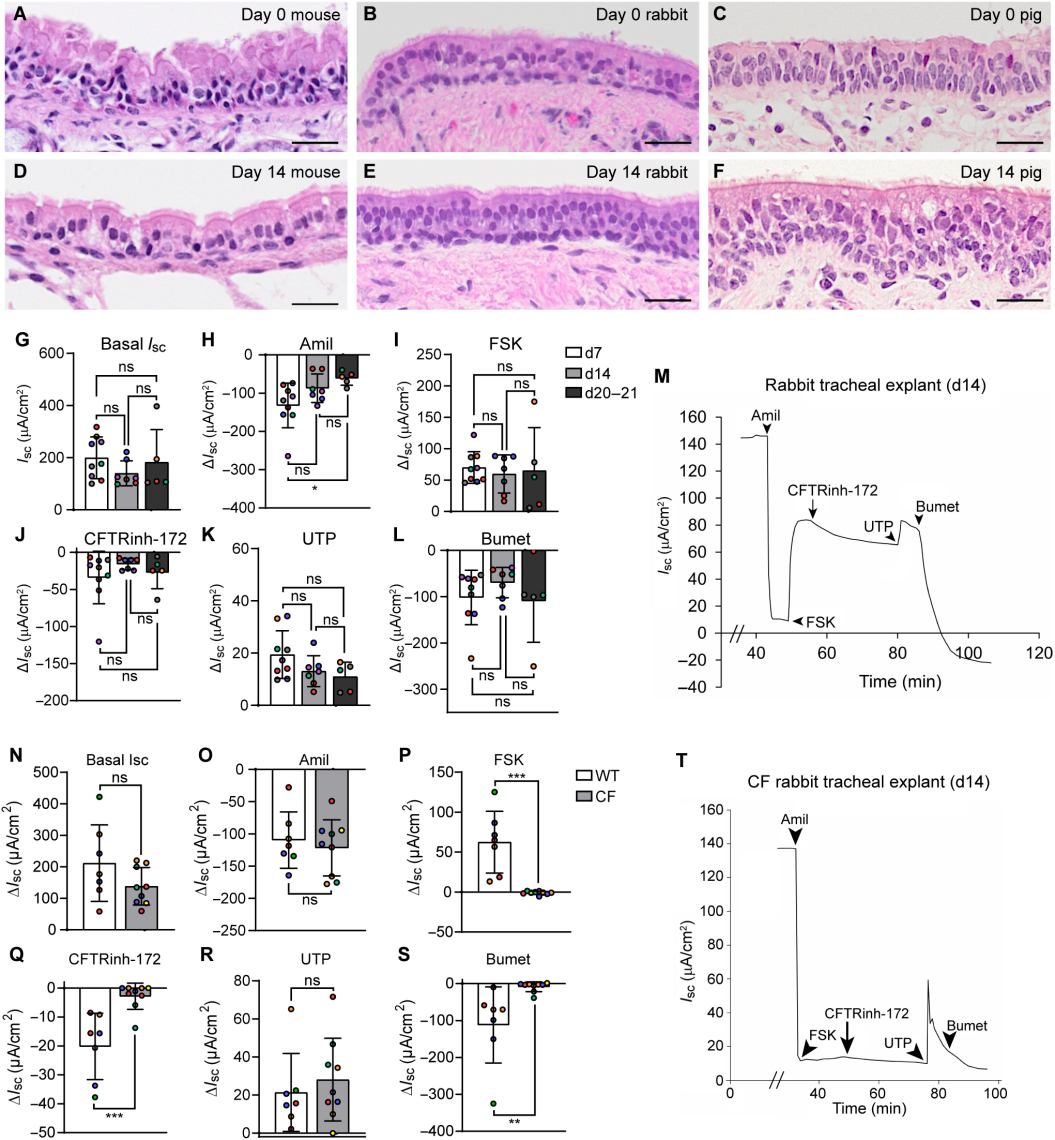
Viable airway explants can be made from diverse animal models. (**A** to **C**) H&E histology of day 0 (A) mouse, (B) rabbit, and (C) pig tracheal explants. (**D** to **F**) H&E histology of day 14 (D) mouse, (E) rabbit, and (F) pig tracheal explants. Scale bars, 25 μm. Representative of *n* = 3 mice [(A) and (D)], 3 rabbits [(B) and (E)], and 4 pigs [(C) and (F)]. (**G** to **L**) Time course of rabbit tracheal explant electrophysiology at day 7, day 14, and days 20 and 21. (G) Basal *I*_sc_ and change in Δ*I*_sc_ in response to (H) Amil, (I) FSK, (J) CFTRinh-172, (K) UTP, and (L) bumetanide. *n* = 4 to 6 animals (represented by different colored dots); one to two replicates per animal. (**M**) Representative Ussing tracing of a day 14 rabbit tracheal explant. (**N** to **S**) Electrophysiology of CF versus wild-type rabbit tracheal explants at day 14. (N) Basal *I*_sc_ and Δ*I*_sc_ in response to (O) Amil, (P) FSK, (Q) CFTRinh-172, (R) UTP, and (S) bumetanide. *n* = 4 to 5 animals (represented by different colored dots); one to two replicates per animal. (**T**) Representative Ussing tracing of a CF rabbit tracheal explant at day 14. [(G) to (L)] One-way analysis of variance (ANOVA) with Tukey’s posttest. [(N) to (S)] Unpaired *t* test. *P < 0.05, **P < 0.01, and ***P < 0.001.

To determine whether methods developed for human explants are applicable to animal models, we excised mouse, rabbit, and pig tracheal explants from acutely euthanized animals. Histological examination of mouse, rabbit, and pig tracheal explants after 0 days (Fig. 6, A to C) or 14 days (Fig. 6, D to F) in culture revealed highly ciliated epithelia. The electrophysiology of rabbit tracheal explants was investigated after 7, 14, and 21 days in culture (Fig. 6, G to M), and markedly higher responses to Amil, FSK, and CFTRinh-172 in cultured explants were observed compared to previously published measurements of fresh tissue (*23*). ENaC activity dropped over time (Fig. 6H); however, all other ion transport was preserved (Fig. 6, G and I to L). Comparisons of tracheal explants from wild-type and CFTR knockout rabbits (*23*) revealed the expected decrease in FSK and CFTRinh-172 response in CFTR knockout tissues (Fig. 6, N to T). We also characterized the electrophysiology of pig tracheal explants after 7, 14, and 21 days in culture (fig. S5). In the porcine model, there were trends toward reduced ion transport at day 21 compared to day 7 measurements with a significant difference in the bumetanide response only.

Last, we measured mucociliary clearance (MCC) in rabbit tracheal explants by tracking fluorescently labeled beads aerosolized onto the tissue explant surface. MCC was higher in explants at all time points than in previously reported in situ measurements (*24*) (fig. S6A). This finding may indicate that rabbit tracheal explants have more cilia per unit area than the in vivo rabbit trachea, reflecting ~30% shrinking of explant length upon removal from the animal (fig. S6B).

### Airway tissue explants created from cryopreserved tissue

A challenge of the human airway explant model is dependence on the availability of human tissue. Cryopreservation of human airway tissue would allow investigators to store freshly dissected airway tissues for use on demand. To determine whether viable airway explants could be prepared from cryopreserved tissue, we froze freshly excised human bronchial segments using the CryoStor commercial freezing solution and stored them in liquid nitrogen for days to months. Upon thawing, tissues were rinsed in phosphate-buffered saline (PBS), dissected as previously, and placed on Gelfoam for culture.

**Fig. 7.**
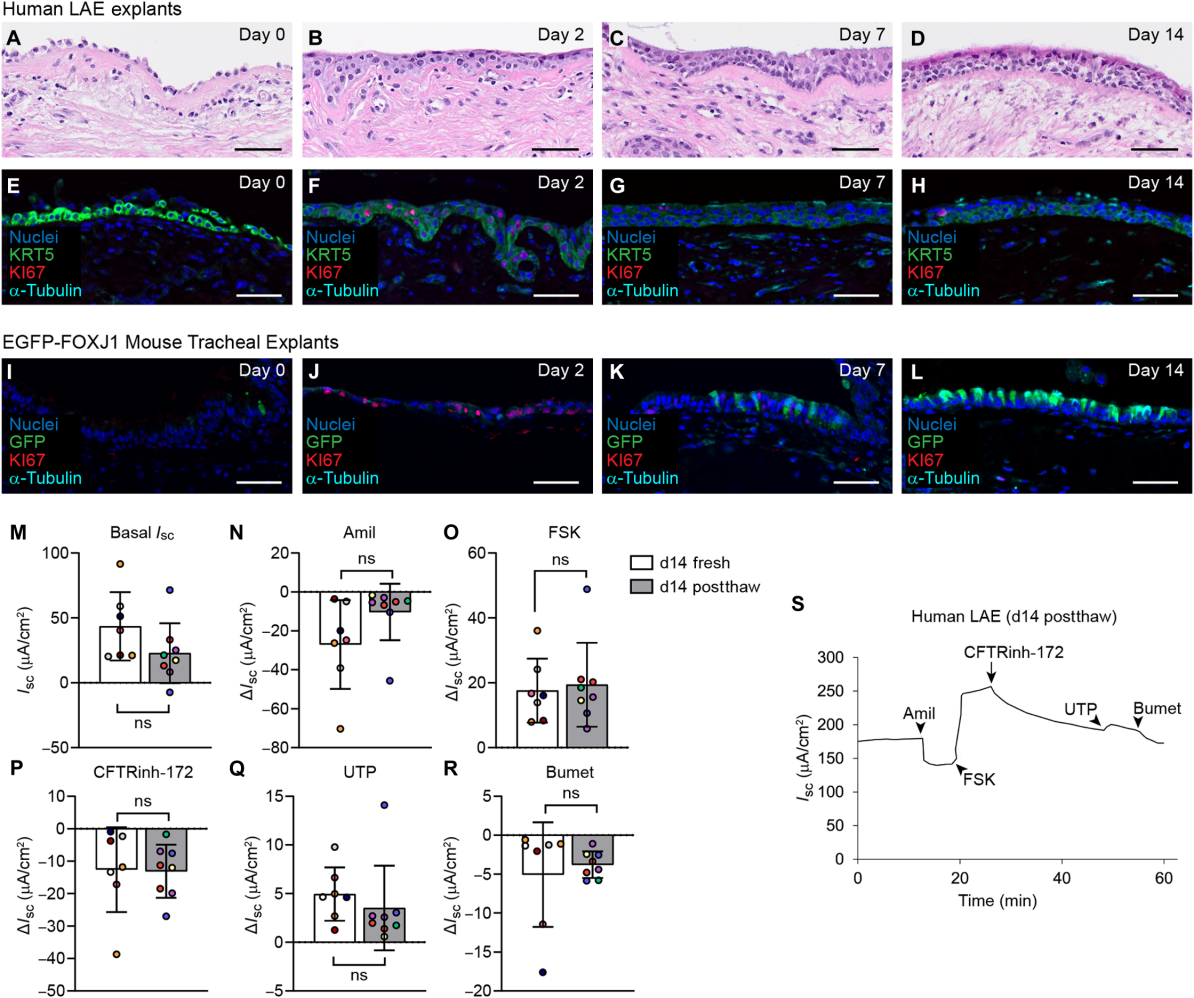
Airway explants can be made from cryopreserved tissue. (**A** to **D**) H&E histology of cryopreserved/thawed human LAE explants at day 0 (A), day 2 (B), day 7 (C), and day 14 (D) postthaw. Scale bars, 50 μm. (A to D) Representative of *n* = 3 donors. (**E** to **H**) IF localization of KRT5, KI67, and α-tubulin in cryopreserved/thawed human LAE explants at day 0 (E), day 2 (F), day 7 (G), and day 14 (H) postthaw. Scale bars, 50 μm. [(E) to (H)] Representative of *n* = 3 donors. (**I** to **L**) IF localization of EGFP-FOXJ1 mouse tracheal explants made from cryopreserved tissue at day 0 (I), day 2 (J), day 7 (K), and day 14 (L) postthaw. Scale bars, 50 μm. [(I) to (L)] Representative of *n* = 3 mice. (**M** to **R**) Electrophysiology of human LAE explants made from fresh tissue at day 14 or cryopreserved tissue at day 13 and day 14 postthaw. (M) Basal *I*_sc_. Change in Δ*I*_sc_ in response to (N) Amil, (O) FSK, (P) CFTRinh-172, (Q) UTP, and (R) bumetanide. *n* = 5 to 6 donors (represented by different color dots); one to two replicates per donor. (**S**) Representative Ussing tracing of a human LAE explants made from cryopreserved tissue at day 14 postthaw. [(M) to (R)] Unpaired *t* test.

Frozen/thawed LAEs exhibited a sparse basal cell layer immediately after thawing, but epithelial cells covered the surface by day 2 postthaw (Fig. 7, A to B). At day 7, the thawed epithelium was pseudostratified but poorly ciliated, with cilia returning by day 14 (Fig. 7, C to D). IF staining confirmed that the epithelial layer was nearly 100% KRT5^+^ basal cells at day 0, which proliferated at day 2 and differentiated into α-tubulin^+^ ciliated cells by day 14 (Fig. 7, E to H). To further investigate the kinetics of cell renewal, we created airway explants from cryopreserved tracheal tissues from transgenic mice carrying an enhanced green fluorescent protein (EGFP) tag under the FOXJ1 promoter (EGFP-FOXJ1) (*25*). In this model, FOXJ1^+^ ciliated cells are marked by EGFP expression. EGFP expression was lost in the first 2 days after thawing but reemerged by day 7, following increased KI67 expression in basal cells at day 2 (Fig. 7, I to L). From these data, we conclude that ciliated cells are lost upon airway cryopreservation but regenerate from basal cells during culture on Gelfoam. Last, the function of frozen/thawed human airway explants was assessed by Ussing chamber measurements at day 14 postthaw. The electrophysiology of frozen/thawed LAEs was similar to never-frozen explants (Fig. 7, M to S). This finding was confirmed in frozen/thawed rabbit tracheal explants (fig. S6, C to K). Thus, we concluded that airway tissue explants generated from cryopreserved human or animal airway tissues regain key morphologic and electrophysiologic features after 14 days in culture.

### Human airway explants model viral tropism and host gene responses

Study of viral infection in human airway tissue explants permits study in an in vivo–like environment with conserved tissue architecture and cells from diverse lineages. Because ex vivo human tissues have been historically limited in longevity, prior studies required viral inoculation on the same day as tissue harvest (*26*–*28*). The practicality of airway explant models would be greatly expanded if tissues could be infected after days or weeks in culture.

**Fig. 8.**
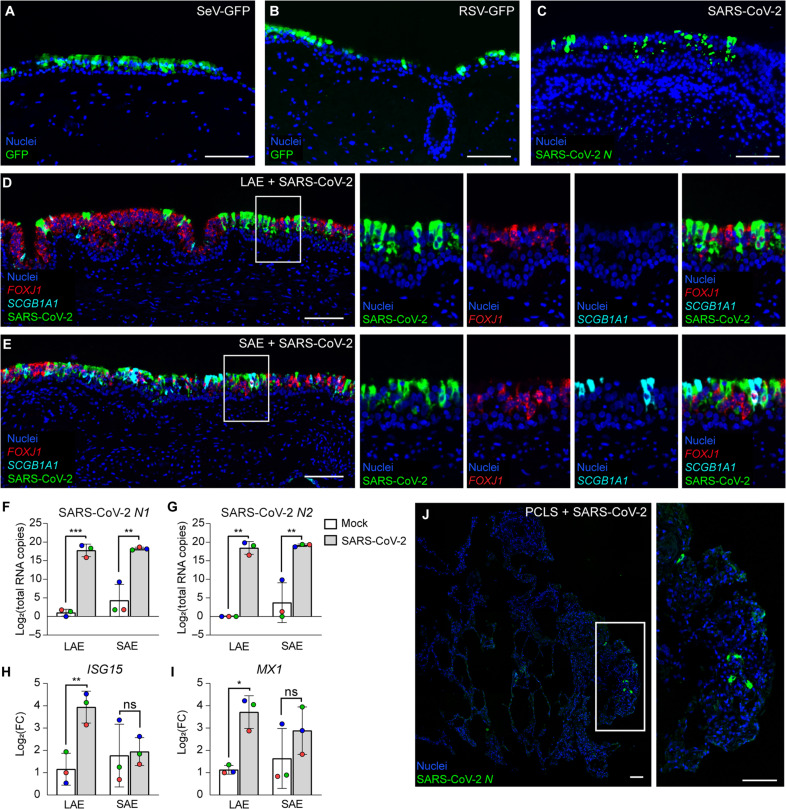
Viral infection in human LAE, SAE, and PCLS models. (**A** to **C**) Viral infection of human LAEs with (A) SeV-GFP after 45 days in culture, (B) RSV-GFP after 45 days in culture, or (C) the D614G variant of SARS-CoV-2 after 29 days in culture. IF localization of GFP (A and B) or SARS-CoV-2 (C) at 4 dpi. Scale bars, 100 μm. [(A) to (C)] Representative of *n* = 2 donors; two replicates per donor. (**D** and **E**) Fluorescent RNA in situ hybridization of human LAEs (D) and SAEs (E) inoculated with the D614G variant of SARS-CoV-2 after 25 days in culture. Stained at 4 dpi for *FOXJ1* (red), *SCGB1A1* (cyan), and *SARS-CoV-2* (green). Nuclei counterstained with 4′,6-diamidino-2-phenylindole. Scale bars, 100 μm. [(D) and (E)] Representative of *n* = 3 donors. (**F** and **G**) Log base 2 of the relative copy number of SARS-CoV-2 nucleocapsid genes *N1* (F) and *N2* (G) measured by quantitative real-time polymerase chain reaction (qRT-PCR) and compared to a standard curve in human LAE and SAE explants at 3 dpi. *n* = 3 donors; one replicate per donor. (**H** and **I**) Log base 2 of the relative gene expression of interferon stimulated genes *ISG15* (H) and *MX1* (I) measured by qRT-PCR in human LAE and SAE explants at 3 dpi. The fold change (FC) in gene expression was normalized to *TBP* expression. *n* = 3 donors; one replicate per donor. (**J**) Representative fluorescent RNA in situ hybridization of human PCLSs inoculated with the D614G variant of SARS-CoV-2 after 7 days in culture. Scale bars, 100 μm. Representative of *n* = 4 donors. [(F) to (I)] Data were log transformed before statistical testing due to unequal variances among samples and analyzed using a linear mixed-effects model with the donor as a random effect factor. *P < 0.05, **P < 0.01, and ***P < 0.001.

A panel of viruses was studied in human LAEs including a Sendai virus expressing GFP (SeV-GFP), a respiratory syncytial virus expressing GFP (RSV-GFP), and the D614G variant of severe acute respiratory syndrome coronavirus 2 (SARS-CoV-2) after 18 to 45 days in culture. RSV and SARS-CoV-2 are known to cause respiratory disease in humans (*29*, *30*). Although SeV is host restricted to rodents and does not typically cause human disease, it has been proposed as a live virus vaccine vector, a potential gene therapy vector, and infection has been studied in human bronchial epithelial cells in vitro (*31*), but not in human explant models. Robust infection was observed at 4 days postinfection (dpi) with all studied viruses as indicated by IF localization of GFP (Fig. 8, A and B) or SARS-CoV-2 nucleocapsid (Fig. 8C).

More detailed studies focused on the SARS-CoV-2 infection given the importance of the recent COVID-19 pandemic. Human LAEs and SAEs were inoculated with SARS-CoV-2 and assessed for viral tropism by fluorescent RNA in situ hybridization at 4 dpi. As expected, SARS-CoV-2 nucleocapsid transcript was predominantly expressed in FOXJ1^+^ ciliated cells in both LAE and SAE tissues (Fig. 8, D and E). We then quantified RNA copy numbers of SARS-CoV-2 nucleocapsid genes *N1* and *N2* in infected LAEs and SAEs by performing quantitative real-time polymerase chain reaction (qRT-PCR) (Fig. 8, F and G). High levels of *N1* and *N2* RNAs were present in both LAEs and SAEs at 3 dpi but were undetectable in mock-infected LAE and SAE cultures. The interferon-stimulated genes (ISGs), *ISG15* and *MX Dynamin-Like GTPase 1* (*MX1*), were also elevated at 3 dpi, indicating that airway tissue explants are not only infectable but can also respond to viral infection by activating interferon-mediated host innate defense pathways (Fig. 8, H and I). Notably, the infection rate and ISG response were indistinguishable between airway tissues inoculated 1 to 2 days after dissection and airway explants cultured for 16 to 24 days before infection (fig. S7, A to D). Viral infection was also observed in airway explants generated from cryopreserved tissue (fig. S7E).

Last, to establish an ex vivo infection model in the distal lung, we infected human PCLS tissues with the D614G variant of SARS-CoV-2 after 7 days of culture on Gelfoam. Infection was less robust in the alveolar epithelium modeled by PCLSs compared to LAEs and SAEs, aligning with previous reports using cultured airway cells and distal lung organoids (Fig. 8J) (*32*). From these data, we concluded that human airway explants and PCLSs can be used to study viral infection after several weeks in culture.

## DISCUSSION

The profound worldwide impact of emerging respiratory tract pathogens highlights the vital importance of physiologically relevant models of the airway epithelium and distal lung. The Gelfoam-ALI airway explant model preserves characteristic tissue architecture and diverse cell lineages for at least 14 days in culture. Cell populations within the explant models are dynamic, with certain quiescent cell populations lost (Fig. 5, A, Q, and S) and repairing cell populations gained (Fig. 5, B to D and Z). Nonetheless, airway explants provide a unique platform for studying epithelial-stromal and epithelial-immune cell interactions that cannot be studied by standard in vitro methods.

Previous studies have measured airway epithelial electrophysiology immediately after surgical resection (*8*, *17*). Here, airway explants displayed no measurable electrophysiology when studied immediately after dissection, but we suspect that this may be due to the long cold ischemic time of our studied tissues. Even so, functional ion transport recovered by days 1 and 2 and days 14 and 15 (Fig. 3, A to L) to levels of previously reported fresh tissue measurements (*8*, *17*), indicating a remodeling and/or reoxygenation of airway explants during the Gelfoam culture. A proliferating basal cell population was detected by scRNA-seq (Fig. 5B) and confirmed by IF in day 2 explants (fig. S3E). This population was lost by day 14 (Fig. 5B and fig. S3F), consistent with transient repair, followed by a return to quiescence. These data highlight the utility of the explant model for studying injury and repair ex vivo.

The recovery of airway tissue function after ~40 hours of cold ischemic injury is important, as it greatly increases the number of lungs that can be studied. With time in culture, our scRNA-seq data revealed a subset of basal/suprabasal cells expressing transcripts associated with airway epithelial hypoxia in LAE and SAE explants (*20*). Metabolically active basal cells in large airways in vivo may partially receive O_2_ from submucosal systemic capillaries, and absence of vascular perfusion in explants may contribute to hypoxia. Similarly, the up-regulation of glycolysis enzyme genes was observed in endothelial and stromal cells in day 14 explant cultures. This likely reflects a metabolic shift in these cells, localized in submucosal regions, as they adapt to a relatively hypoxic environment created by culture conditions. In addition, airway epithelia gain O_2_ from the lumen, and accumulated mucus may result in O_2_ limitation. While mucin secretion is a key property of airway epithelia for maintaining MCC, this observation highlights the importance of removing apical mucus from airway explants to maintain epithelial homeostasis over time. Alternatively, airway explants maintained on Gelfoam sponge without mucus removal induce asymmetric apical hypoxia to model muco-obstructive lung diseases, including CF, chronic obstructive lung disease, and asthma.

The ability to prepare viable airway explants from cryopreserved tissue presents another major advancement in the field. While cryopreservation of PCLSs has been previously reported (*33*–*35*), our study represents the application of this methodology to generate viable airway tissue explants from cryopreserved tissue. Airway explants from cryopreserved tissue successfully replicated morphologic and electrophysiologic features of fresh tissue. Although cryopreservation severely injures the airway epithelium (Fig. 7, A to L), KRT5^+^ basal cells were retained and regenerated a viable, ciliated epithelium. Future scRNA-seq studies to characterize other cellular changes in the cryopreservation recovery process should be informative for understanding mechanisms of airway regeneration. In vitro, airway basal cells can be efficiently transduced by gene transfer vectors for genetic modification (*36*, *37*). Thus, unobstructed access to the basal cell layer following cryo-injury could facilitate gene modulation in ex vivo human tissues, a unique application of this technology.

The ability to culture viable explants from diverse animal models could improve translation between animal and human studies. Further, the ability to generate multiple explants per animal and the ability to cryopreserve excess tissue for later experiments aligns well with the “three Rs principle of animal research,” replacement, reduction, and refinement (*38*). Unlike in vivo experiments where only one condition can be tested per animal, explant culture allows airway tissue to be divided into multiple explants, e.g., 20 or more explants per adult rabbit trachea, permitting the study of multiple conditions and/or replicates per animal. Another advantage is the ability to create explant cultures from animal tissues carrying genetic modification. This feature was demonstrated here through the use of CFTR knockout rabbits (*23*) and EGFP-FOXJ1 transgenic mice (*25*).

The Gelfoam-cultured airway explants can be used to study viruses including SeV, RSV, and SARS-CoV-2. SARS-CoV-2 tropism in the airway explant was comparable to COVID-19 autopsy samples indicating ciliated cells as the primary target of viral entry in the airways (*32*) with lower infection rates in the distal lung (*31*). Previous groups have studied viral infection in ex vivo tissue models (*26*, *39*) including PCLSs (*40*, *41*). However, all prior work was restricted to short-term time points and required inoculation immediately after tissue procurement. In our study, airway tissue explants cultured on Gelfoam could be infected after 18 to 45 days in culture, and response to infection was nearly identical to inoculated fresh tissues (fig. S7, A to D). The ability to infect airway tissue explants after extended culture or following cryopreservation (fig. S7E) greatly expands this model’s practicality. This feature also gives airway tissue explants time to recover from cold ischemia and dissection before viral inoculation. Here, we studied viral infection at 4 dpi, but studies of late-stage recovery from viral infection are a future application of this work.

The current form of the Gelfoam-explant model has limitations: Rabbit tracheal explant electrophysiology differed from published measurements of fresh rabbit trachea (*23*), with substantially higher Amil-, FSK-, and CFTRinh-172–sensitive currents than fresh tissues (Fig. 6, G to L). Pig tracheal explant electrophysiology also differed from previously published measurements of freshly excised tissue (*42*, *43*), with a greater basal *I*_sc_ and a greater FSK response in our day 14 pig tracheal explant model. Future work will be required to identify the mechanisms underlying enhanced ion transport in the rabbit and pig explant model and why these differences were not observed in the human explant model. We also observed that relatively thinner airway explants, e.g., rabbit tracheal explants, exhibited gradual shrinkage from the edge over time in culture (fig. S6B). This observation underscores the need for further optimization of explant culture methods for thinner airway explant tissues. Further, although the in vivo alveolar space is typically quiescent, high proliferation rates in various cellular compartments were observed in the day 14 PCLS model (Fig. 2, G to H). This proliferation may be a response to the mechanical injury incurred during PCLS preparation (tissue slicing on a Compresstome). Alternatively, the prolonged proliferation could point to innate differences in culture requirements or differences in how the airway and alveolar epithelium respond to the material properties and mechanical cues of Gelfoam. Although Gelfoam-culture prolonged PCLS viability considerably (Fig. 2C), further medium optimization and exploration of different matrix substrata may be warranted.

In summary, we have shown that culture of human airway explants and PCLSs at ALI on a Gelfoam sponge prolongs tissue viability for several weeks (a summary of preserved tissue properties can be found in table S2). Explant models have distinct advantages over in vitro models by preserving diverse organotypic cell types and are thus well suited for future studies of epithelial-stromal and epithelial-immune cross-talk. These models also allow for direct comparison of anatomically distinct lung regions (i.e., large airway, small airway, and alveolar parenchyma) and could provide future insights into the coordination that exists between these connected but regionally distinct lung compartments. By extending the viability of airway explants beyond previously established methods, the Gelfoam explant model may enable future studies of chronic or repeated injury, induction and reversal of fibrosis, or the long-term responses to viral infection. These methods are generally applicable to animal models including mouse, rabbit, and pig. Airway tissue explants created from cryopreserved tissue mimic fresh explants, and airway tissue explants can be used to model viral infection and host gene response. Overall, the Gelfoam-ALI explant model expands the practicality of ex vivo airway tissue studies and could enable many important future applications.

## MATERIALS AND METHODS

### Experimental design

The objective of the study was to develop long-term culture methods for human and animal airway explants. Toward this end, human airway tissue, human PCLS, and animal tracheal tissue were prepared and cultured on a Gelfoam sponge as described below.

### Study approval

Protocols for informed consent and obtaining explanted human lung tissue were approved under the University of North Carolina’s (UNC’s) Office of Human Research Ethics/Institutional Review Board study #03-1396.

### Animal ethics statement

All mouse and rabbit experiments were approved by the UNC Institutional Animal Care and Use Committee and performed in accordance with the guidelines and regulations governing the use of these laboratory animals (protocol numbers 20-264.0 and 21-188.0). Pig experiments were approved by the North Carolina State University Institutional Animal Care and Use Committee and performed in accordance with the guidelines and regulations governing the use of these laboratory animals (protocol number 21-258-B).

### Preparation of Gelfoam and airway tissue dissection

Gelfoam (Ethicon, 1972) was cut using sterile scissors into ~1 cm–by–1 cm squares and was placed in a six-well plate (Corning, #3516). Explant medium, prepared by adding l-glutamine (Gibco, #25030-081), penicillin-streptomycin (Gibco, #15140-122), and 10% heat-inactivated fetal bovine serum (Gibco, #16140-071) to Dulbecco’s modified Eagle medium, high glucose (DMEM-H; Gibco, #11965-092), was dispensed into the plate at 3 ml per well. The Gelfoam was rehydrated by gently pressing the Gelfoam sponge into explant medium using ethanol-sterilized forceps. A cocktail of supplemental antibiotics and antifungals including amphotericin B (1 μg/ml), ceftazidime (100 μg/ml), tobramycin (80 μg/ml), vancomycin (100 μg/ml), mycamine (20 μg/ml), and fluconazole (25 μg/ml) was added during the first 24 hours of culture. Additional antibiotics and/or antifungals were added for CF tissues as before (*44*) according to the patient’s clinical records. The explant medium surrounding the Gelfoam was removed and replaced every 2 to 3 days.

LAEs were obtained from first- to third-generation bronchi from previously healthy deceased lung donors or from CF transplant lungs. To prepare LAEs, a 1-cm segment of unbranched bronchus was removed and cut longitudinally to expose the airway lumen. The surface epithelium was then stripped away from the underlying cartilage using surgical microscissors under a dissecting microscope. SAEs were obtained from bronchioles with diameter less than 2 mm. Bronchioles were microdissected from the surrounding parenchyma as previously described (*45*) and cut longitudinally to expose the airway lumen. After dissection, LAEs and SAEs were placed on a medium-soaked Gelfoam sponge with the lumen exposed to air and maintained in a high-humidity incubator. Mouse, rabbit, and pig tracheal explants were prepared by the same methods except that the mouse tracheal epithelium was left attached to the underlying cartilage. Rabbits were anesthetized with isoflurane and exsanguinated by severing the abdominal aorta. Mice were euthanized by CO_2_ inhalation, and pigs were euthanized by barbituric IV injection following sedation. We studied female wild-type and EGFP-FOXJ1 transgenic mice on a mixed C3H × C57BL/6 background (*25*), wild-type and CFTR knockout New Zealand White rabbits (*23*), and wild-type American Yorkshire × Duroc pigs. A detailed protocol describing airway explant preparation and culture can be found in the Supplementary Materials.

### Preparation of PCLSs

Human PCLSs were prepared from previously healthy donor lungs as previously described (*10*, *46*–*48*). Briefly, a piece of distal lung was isolated, warmed to room temperature, and inflated using a needle and syringe with warm (42°C) 3% low-melting point agarose (Thermo Fisher Scientific, #BP165-25) diluted in DMEM-H (Gibco, #11965-092). The inflated piece of lung was then cooled to 4°C for at least 30 min to solidify the agarose. An Acu-punch Biopsy Punch (Thermo Fisher Scientific, #NC9324386) was used to cut 8-mm cores from the inflated lung. Cores were glued to the end of a specimen tube plunger using Scotch super glue (3M, #AD119) and loaded onto a Compresstome (Precisionary) where the tissue was cut to 300 μm in thickness. PCLSs were then placed on Gelfoam for long-term culture and monitored using a WST-8 cell counting kit (VWR, #89155-898) according to the kit’s instructions. Briefly, PCLSs were moved to a 24-well plate using forceps and submerged in 500 μl of WST-8 assay medium for 1 hour at 37°C. The PCLSs were then returned to Gelfoam or submerged culture conditions, and samples of the WST-8 assay medium were transferred to a 96-well assay plate for colorimetric reading using a FLUOstar Omega microplate reader (BMG LABTECH).

### Electrophysiology measurements

*I*_sc_ recordings in Ussing chambers were conducted on human LAEs and SAEs, rabbit tracheal, and pig tracheal explants according to previously published protocols (*23*, *49*). Human, rabbit, and pig airway tissues were mounted with an exposed surface area of 0.025, 0.094, and 0.053 cm^2^, respectively. Both sides of the airway explant were perfused with equal amounts (10 ml) of standard KBR solution (140 mM Na^+^, 120 mM Cl^−^, 5.2 mM K^+^, 1.2 mM Mg^2+^, 1.2 mM Ca^2+^, 2.4 mM HPO_4_^2−^, 0.4 mM H_2_PO_4_^−^, and 25 mM HCO_3_^−^) at 37°C and gassed with 95% O_2_ and 5% CO_2_, providing gas lift circulation. Samples were equilibrated under voltage clamp (*I*_sc_) conditions for at least 20 min until a baseline *I*_sc_ was achieved. Drugs were added as follows: Amil (apical, 10 μM), FSK (basolateral, 10 μM), CFTRinh-172 or GlyH-101 as indicated (apical, 10 μM), uridine 5′-triphosphate (UTP) (apical, 100 μM), and bumetanide (basolateral, 1 mM).

### Mucociliary clearance

To measure MCC, fluorescently labeled beads (200 nm) were aerosolized onto the surface of airway tissue explants, visualized under a dissecting microscope, and recorded, as previously (*24*).

### Immunostaining and RNA in situ hybridization

Explant tissues were fixed by submersion in 10% neutral-buffered formalin for 2 to 7 days, washed using PBS, and stored in 70% ethanol before embedding and serial sectioning. Formalin-fixed paraffin-embedded (FFPE) sections were stained with H&E and AB-PAS. FFPE sections were also assessed by IF localization as previously described (*50*) (see table S3 for antibodies and dilutions). Colorimetric or fluorescent RNA in situ hybridization was performed as previously described (*45*) (see table S4 for a list of materials).

### Cryopreservation and thawing of airway tissue explants

Small (~1-cm) segments of bronchus or microdissected bronchioles were isolated and transferred to cryovials containing 1 ml of CryoStor freezing medium (MilliporeSigma, #C2874) as intact airways. Cryovials were stored in a Corning CoolCell Freezing Container (Corning, #432000) and subsequently frozen at −80°C. After 48 to 72 hours, cryovials were then transferred to liquid nitrogen for longer term storage. To thaw, cryovials were incubated in a 37°C water bath until all CryoStor freezing medium was melted. Airway tissue was then rinsed in PBS, cut longitudinally to expose the airway lumen, and placed on a Gelfoam sponge for long-term culture. PCLS tissue cut using a Compresstome were frozen and thawed in the same manner.

### Single-cell dissociation of LAE, SAE, and PCLS tissues

LAE, SAE, and PCLS tissues were cryopreserved in CryoStor freezing medium after 0 (fresh), 2, and 14 days in culture. Cryovials were thawed, rinsed in PBS, placed into a 15-ml tube containing 5 ml of Accutase solution (Sigma-Aldrich, #A6964-100ML) with EDTA (5 mM; Invitrogen, #15575-038) and pronase (1 mg/ml; Roche, #10165921001), and incubated on a shaker for 30 min at 37°C. Following incubation, the tube was centrifuged (600*g* for 2 min at 4°C), the supernatant was discarded, and 5 ml of Hank’s balanced salt solution (Ca^−^, Mg^−^) (Gibco) buffer containing liberase (50 mg/ml; Roche, #05401119001) and deoxyribonuclease (25 μg/ml; Roche, #10104159001) was added to the tube. The sample was then incubated on the shaker for 10 min at 37°C. After incubation, the cell suspension was gently pipetted 20 times using a wide-orifice pipette tip. To neutralize enzymes, 500 ml of fetal bovine serum was added to the cell suspension, and the neutralized mixture was filtered through a 100-μm filter, followed by centrifugation (600*g* for 2 min at 4°C). After removing supernatant, dissociated cells were resuspended in PBS (Ca^−^, Mg^−^) containing 0.01% ultrapure bovine serum albumin (Invitrogen, #AM2616), filtered through a 40-μm cell strainer (Bel-Art, H13680-0040), and centrifuged (600*g* for 2 min at 4°C). Cell count and viability were assessed using trypan blue dye exclusion with a Countess 3 automated cell counter (Thermo Fisher Scientific), and the cell concentration was adjusted to approximately 1000 cells/μl. The dissociated cells were used for 10x Genomics scRNA-seq library preparation.

### Chromium 10x Genomics scRNA-seq library preparation and sequencing

Single cells were captured using a 10x Chromium controller (10x Genomics), and libraries were prepared following the Single-Cell 3′ Reagent Kits v3.1 (Dual Index) User Guide (10x Genomics). Cellular suspensions were loaded on a Chromium Controller instrument (10x Genomics) to generate single-cell Gel Bead-In-EMulsions (GEMs). Reverse transcription (RT) was performed in a Veriti 96-well thermal cycler (Thermo Fisher Scientific). After RT, GEMs were harvested, and the cDNA underwent size selection with SPRIselect Reagent Kit (Beckman Coulter). Indexed sequencing libraries were constructed using the Chromium Single-Cell 3′ Library Kit (10x Genomics) for enzymatic fragmentation, end-repair, A-tailing, adapter ligation, ligation cleanup, sample index PCR, and PCR cleanup. Barcoded sequencing libraries were quantified by Agilent TapeStation. The libraries were loaded onto a NovaSeq 6000 (Illumina) with a custom sequencing setting of paired-end reads (28 and 90 cycles for read 1 and read 2, respectively) to achieve a sequencing depth of at least ~5 × 10^4^ reads per cell.

### scRNA-seq data analysis

Sequencing data in FASTQ format per sample were processed and mapped to recent reference human genome and Gencode gene annotation using Cell Ranger v7 pipeline from 10x Genomics. The resultant gene × cell matrices were imported into R using the Seurat package (*51*) after ambient RNA contamination adjustments using R package, SoupX (*52*), with recommended parameters. Cells were filtered at >300 gene counts, maximal gene counts varying between 7000 and 9000 gene counts, and <20% mitochondrial gene expression. The samples were integrated into a combined dataset using the IntegrateData function from Seurat with targeted 4000 anchor genes and 50 principal components analysis (PCA) dimensions. Cell clusters from the integrated dataset were generated with the FindClusters function with 40 PCA dimensions and a resolution parameter of 0.5. Cell type predictions were made using local setup of azimuth (https://azimuth.hubmapconsortium.org/) against the core human lung cell atlas version 2 from normal lung samples (*18*). Final cell type labels were assigned manually on the basis of marker genes analysis with FindAllMarkers function from Seurat and predicted cell types. Cell cluster differential abundance testing was performed on sample-wise cell count matrix using the Bioconductor package edgeR with generalized linear model (GLM) fit, negative binomial (NB) distribution, and quasi-likelihood (QL) *F* test (*53*, *54*). Differential gene expression analysis was performed using the hurdle method (*55*) on normalized gene expression data. The hurdle method combines a GLM test of positive cell proportions and linear model of mean difference in log-normalized expression among positive cells between different groups. Various plots were generated with Seurat and the R package, ggplot2 (*56*).

### Quantitative real-time PCR

Total RNA from explant cultures was extracted with TRI reagent (Sigma-Aldrich, T9424) and an RNA extraction kit (Zymo Research, R2052) according to the manufacturer’s instructions. Total RNA was reverse transcribed to cDNA using the iScript cDNA Synthesis Kit (Bio-Rad, 1708890). qRT-PCR was performed using TaqMan probes, including *2019-nCoV_N1* (IDT, 10006775), *2019-nCoV_N2* (IDT, 10006776), *ISG15* (Hs01921425_s1), and *MX1* (Hs00895608_m1), with SsoAdvanced Universal Probes Supermix (Bio-Rad, 172-5284) using QuantStudio6 Real-time PCR machine (Applied Biosystems). For the calculation of ΔΔ*C*_t_ values, undetectable values were assigned *C*_t_ = 40, corresponding to the maximum number of amplification cycles.

### Viral inoculation

Airway tissue explants and PCLSs were removed from the Gelfoam sponge and transferred to an empty well of a 12-well plate (Corning, #3513) using sterile forceps. Explants were then submerged in a virus-containing inoculum and incubated for 2 hours at 37°C. For SARS-CoV-2 inoculation, samples were submerged in 300 μl of virus (1.91 × 10^7^ plaque-forming units) in a 24-well plate. Samples were submerged in 100 μl of virus in a 48-well plate for Sendai virus [0.8 log_10_ median tissue culture infectious dose (TCID_50_)/ml] and RSV (0.65 log_10_ TCID_50_/ml) inoculations. After rinsing with PBS, explants were transferred back to the Gelfoam sponge and cultured for 3 to 4 days. Gene expression of *ISG15*, *MX1*, and SARS-CoV-2 nucleocapsid genes, *N1* and *N2*, was assessed by qRT-PCR. For *N1* and *N2*, the *C*_t_ values were compared to a standard curve to obtain the viral copy number.

### Statistical analysis

Data were analyzed using GraphPad Prism 9.5 (RRID:SCR_002798) by an unpaired *t* test for single comparisons or a one-way analysis of variance (ANOVA) with Tukey’s or Dunnett’s posttest for multiple comparisons or in R Studio (RRID:SCR_000432) using a linear mixed-effects model with the donor as a random effects factor or a QL *F* test as indicated in the figure legends. All data are presented as means ± SD.
