# Supplementary material for: Prolonged airway explant culture enables study of health, disease, and viral pathogenesis
Source: Sci Adv. 2025 Apr 25;11(17):eadp0451. doi: 10.1126/sciadv.adp0451 (PMC12024639; doi:10.1126/sciadv.adp0451)
Supplement: Supplementary file 1 — Supplementary Text Figs. S1 to S7 Tables S1 to S20 [file sciadv.adp0451_sm.pdf]

Science Advances
AAAS

Supplementary Materials for

Prolonged airway explant culture enables study of health, disease, and viral pathogenesis

Rhianna E. Lee-Ferris *et al.*

Corresponding author: Scott H. Randell, scott_randell@med.unc.edu

Sci. Adv. **11**, eadp0451 (2025)
DOI: 10.1126/sciadv.adp0451

This PDF file includes:

Supplementary Text
Figs. S1 to S7
Tables S1 to S20

Airway explant and PCLS protocols

A. Prepare explant media according to the table below.

**Media component**	**Volume added**	**Final concentration**
Dulbecco's Modified Eagle Medium, high glucose (Gibco #11965-092)	440 ml	-
L-Glutamine (Gibco #25030-081)	5 ml	1x
Penicillin-streptomycin (Gibco #15140-122)	5 ml	1x
Heat inactivated fetal bovine serum (Gibco #16140-071)	50 ml	10%
Total	***500 ml***	

B. Prepare Gelfoam® (Ethicon 1972) sponge.

1. Cut a 1cm x 1cm square of Gelfoam® using sterile scissors in a sterile tissue culture hood.
2. Place Gelfoam® squares into a six well plate (Corning #3516).
3. Dispense 3ml explant media into each well of the six well plate.
4. Gently press the Gelfoam® sponge into the media using ethanol-sterlizied forceps. The sponge should fill with media and will change color from opaque white to the color of the media.
5. UV the plate for 5 min to sterilize.

C. Dissection of large airway tissue (LAE) explants.

1. Obtain a 1 cm segment of unbranched airway (first to third generation bronchi).
2. Cut the airway segment lengthwise along the posterior (non-cartilaginous) wall to open the airway tube and expose the airway lumen.
    a. For mouse tracheal explants, the airway was cut a second time along the anterior wall to create two half tubes.
3. Strip the airway tissue away from the underlying cartilage by lifting a corner of the tissue with forceps and cutting with surgical micro-scissors.
    a. For mouse tracheal explants, the cartilage was retained.
4. Using forceps, transfer the airway tissue onto the Gelfoam® sponge with the airway lumen facing the air and the basolateral (cartilage-facing) tissue contacting the Gelfoam®.

D. Dissection of small airway tissue (SAE) explants.

1. Obtain an unbranched segment of bronchiole with diameter <2mm as previously described (*45*).
2. Separate the airway surface epithelium from the underlying parenchyma using surgical micro-scissors and a dissecting microscope.
3. Cut the airway lengthwise to open the airway tube and expose the airway lumen.
4. Using forceps, transfer the airway tissue onto the Gelfoam® sponge with the airway lumen facing the air and the basolateral (parenchyma-facing) tissue contacting the Gelfoam®.

E. *Preparation of human precision cut lung slices (PCLS) as previously* described (*10*, *46–48*).

1. Prepare 3% low-melting point agarose (ThermoFisher #BP165-25) diluted in DMEM-H (Gibco #11965-092) and maintain at 42ºC.
2. Collect a piece of distal lung tissue and bring to room temperature (~30 minutes off ice). Do not let the tissue sit for too long.
3. Using a needle and syringe, inflate the lung tissue with 3% agarose by piercing at different points across the lung tissue.
4. Cool the inflated lung tissue at 4ºC for at least 30 minutes to solidify the agarose.
5. Use an Acu-punch Biopsy Punch (ThermoFisher #NC9324386) to cut 8 mm cores from the inflated lung.
6. Glue the tissue cores to the end of a specimen tube plunger using Scotch super glue (3M #AD119) and load onto a Compresstome® (Precisionary).
7. Slice the tissue to 300 µm thick PCLS.
8. Transfer the PCLS onto freshly prepared Gelfoam® using forceps.

F. Culture of airway tissue explants and PCLS.

1. Add supplemental antibiotics and antifungals for the first 24 hours of culture (see table below). For CF tissues, add additional antibiotics and/or antifungals according to the patient's clinical records as needed (*44*).
2. Replace the explant media surrounding the Gelfoam® every 2-3 days.

**Component**	**Final concentration**
Amphotericin B	1 µg/mL
Ceftazidime	100 µg/mL
Tobramycin	80 µg/mL
Vancomycin	100 µg/mL
Mycamine	20 µg/mL
Fluconazole	25 µg/mL

G. Cryopreservation of airway tissue and PCLS.

1. Obtain a 1 cm segment of unbranched airway or a PCLS as described in the above sections.
2. Transfer the airway segment or PCLS into a cryovial and add 1 mL CryoStor freezing medium (Millipore Sigma #C2874).
3. Place Cryovials in a Corning CoolCell Freezing Container (Corning #432000) and freeze in a -80ºC freezer. The Corning CoolCell Freezing Container will permit a slow freeze of 1ºC per minute.
4. After 48-72 hours, cryovials can then transferred to liquid nitrogen for longer term storage.
5. To thaw, place a cryovial in a 37ºC water bath until all the CryoStor freezing media melts to liquid.
6. Remove the airway tissue or PCLS from the cryovial using ethanol-sterilized forceps.
7. Briefly rinse the tissue with PBS.
8. For cryopreserved airway tissue, dissect airway explants as described above in the '*Dissection of large airway tissue (LAE) explants*' or '*Dissection of small airway tissue*

(SAE) explants' sections and culture according to the '*Culture of airway tissue explants and PCLS'* section.

9. For cryopreserved PCLS, place on a freshly prepared Gelfoam® sponge and culture according to the '*Culture of airway tissue explants and PCLS'* section.

H. Viral inoculation of airway tissue explants and PCLS.

1. Using sterile forceps, remove the airway tissue explant or PCLS from the Gelfoam® sponge and transfer the tissue to an empty well of a 12-well plate (Corning #3513).
2. Submerge the tissue in 300ul of virus-containing inoculum in the 12-well plate.
3. Incubate the tissue for 2 hours at 37ºC.
4. Rinse the tissue with PBS in an empty well of a 12-well plate.
5. Transfer the airway tissue explant or PCLS back to the Gelfoam® sponge using sterile forceps for continued culture.

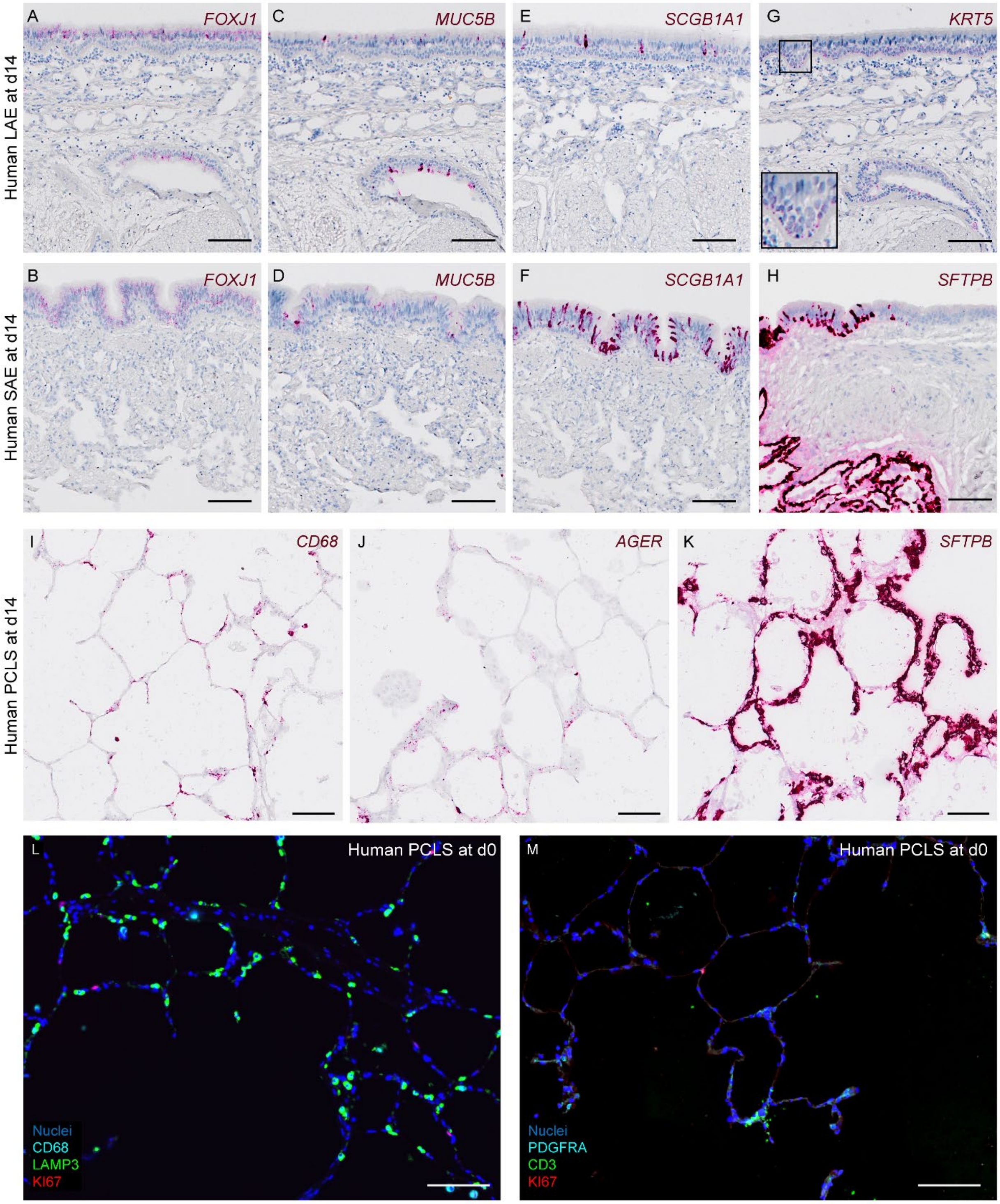

Fig. S1. Human airway explants and PCLS express characteristic markers of epithelial, endothelial, and immune cell populations. (**A-H**) Representative colorimetric RNA *in situ* hybridization images. *FOXJ1* transcript in d14 LAE (**A**) and SAE (**B**) explants. *MUC5B* transcript in d14 LAE (**C**) and SAE (**D**) explants. *SCGB1A1* transcript in d14 LAE (**E**) and SAE (**F**) explants. (**G**) *KRT5* transcript in a d14 LAE explant. (**H**) *SFTPB* transcript in a d14 SAE explant. (**A-H**) Representative of N = 3 donors. (**I-K**) Representative colorimetric RNA *in situ* hybridization for (**I**) *CD68*, (**J**) *AGER*, and (**K**) *SFTPB*. (**I-K**) Representative of N = 3 donors. (**L-M**) Immunostaining of d0 PCLS for CD68, LAMP3, and Ki67 (**L**) and PDGFRA, CD3, and Ki67 (**M**). (**L-M**) Representative of N = 3 donors. All scale bars = 100 μm.

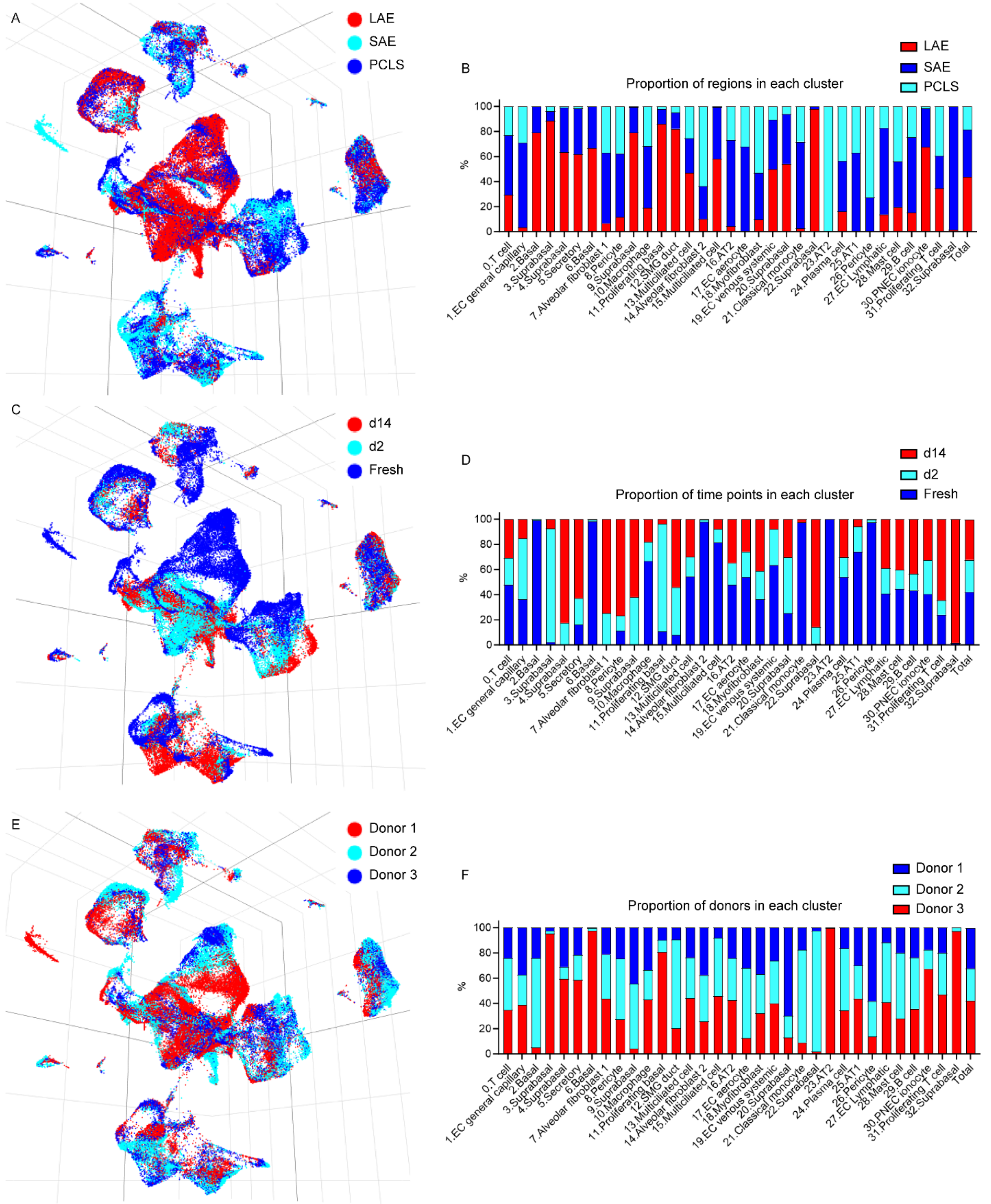

Fig. S2. Single cell RNA-sequencing data shown by region (LAE, SAE, PCLS), time point, and cell donor. (**A**) UMAP colored by region. (**B**) Proportion of the regions that makes up each cluster. (**C**) UMAP colored by time point. (**D**) Proportion of the time points that makes up each cluster. (**E**) UMAP colored by cell donor. (**F**) Proportion of the cell donors that makes up each cluster. (**A-F**) N = 3 donors; 1 replicate per donor.

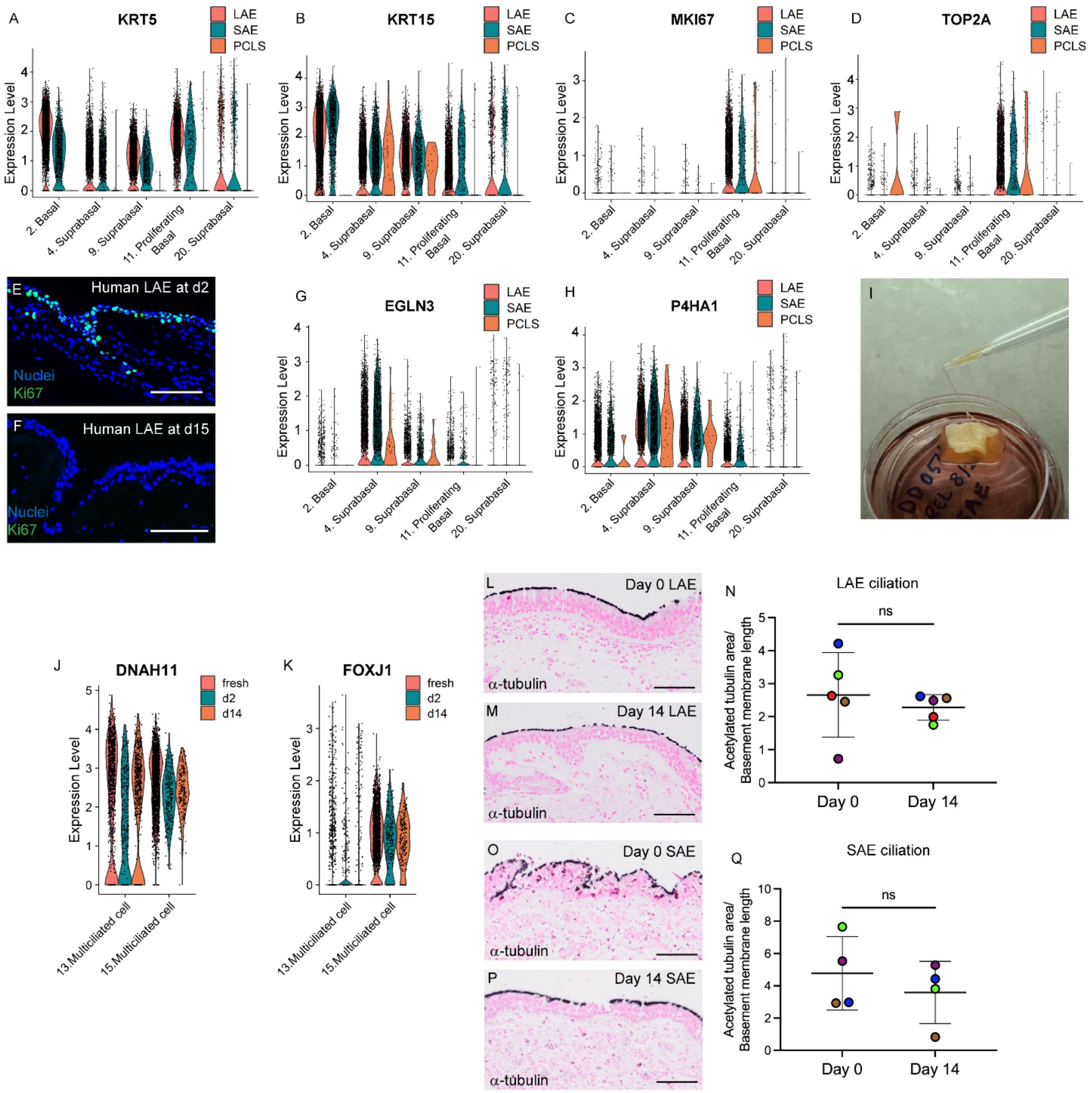

Fig. S3. Basal and suprabasal clusters in the LAE and SAE models. (**A-D**) Violin plot of the basal cell markers, *KRT5* (**A**) and *KRT15* (**B**) and the proliferation markers, *MKI67* (**C**), and *TOP2A* (**D**) in all basal and suprabasal clusters. (**E-F**) Immunofluorescence for KI67 in human LAE at d2 (**E**), and d15 (**F**). (**E-F**) Representative of N=3 donors. (**G-H**) Violin plot of the hypoxia markers, *EGLN3* (**G**) and *P4HA1* (**H**) in all basal and suprabasal clusters. (**I**) Mucus harvested from the apical surface of a human LAE at d28. (**L-J**) Violin plot of the ciliated cell markers, *DNAH11* (**J**) and *FOXJ1* (**K**) in all multiciliated cell clusters. (**L-M**) Immunohistochemistry for a-tubulin to mark ciliated cells in LAE at d0 (**L**) and d14 (**M**). (**L-M**) Representative of N = 5 donors. (**N**) LAE quantitation of a-tubulin area normalized to basement membrane length. N = 5 donors (represented by different colored dots). Paired t-test; ns = non-significant. (**O-P**) Immunohistochemistry for a-tubulin to mark ciliated cells in SAE at d0 (**O**) and d14 (**P**). (**O-P**) Representative of N = 5 donors. (**Q**) SAE quantitation of a-tubulin area normalized to basement membrane length. N = 5 donors (represented by different colored dots). Paired t-test; ns = non-significant.

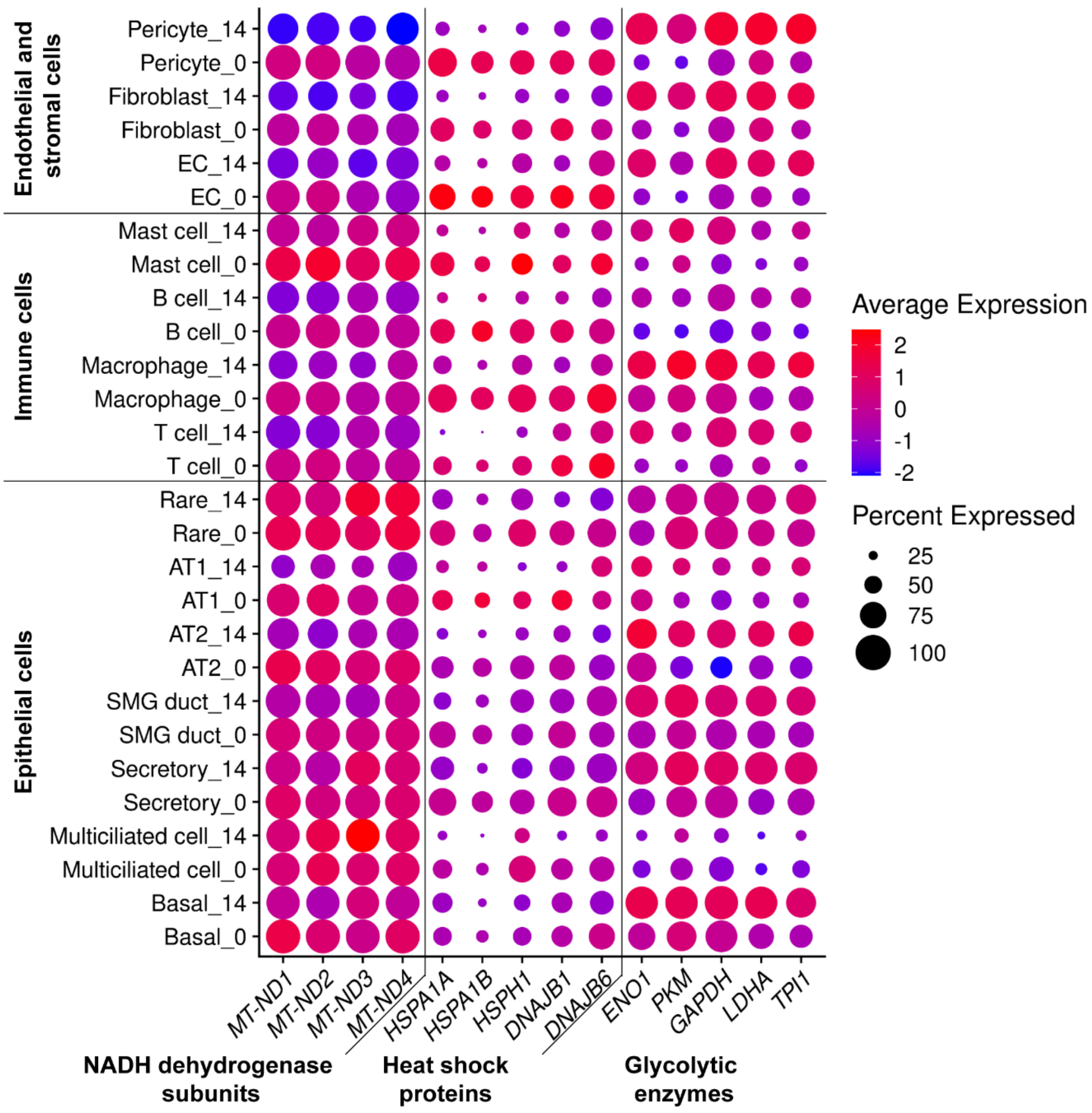

Fig. S4. Common differentially expressed genes across cell types in LAE, SAE, and PCLS models between day 0 and day 14. Common differentially expressed genes (DEGs) between day 0 (fresh) and day 14 samples were defined as those identified by consistently significant (adj.p.value<0.0001 and logFC > 0.1, or adj.p.value<0.0001 and logFC < -0.1) in more than 70% of cell groups (> 10 out of 14). Among 48 common DEGs identified, a subset of genes associated with cellular metabolism and cellular stress is presented.

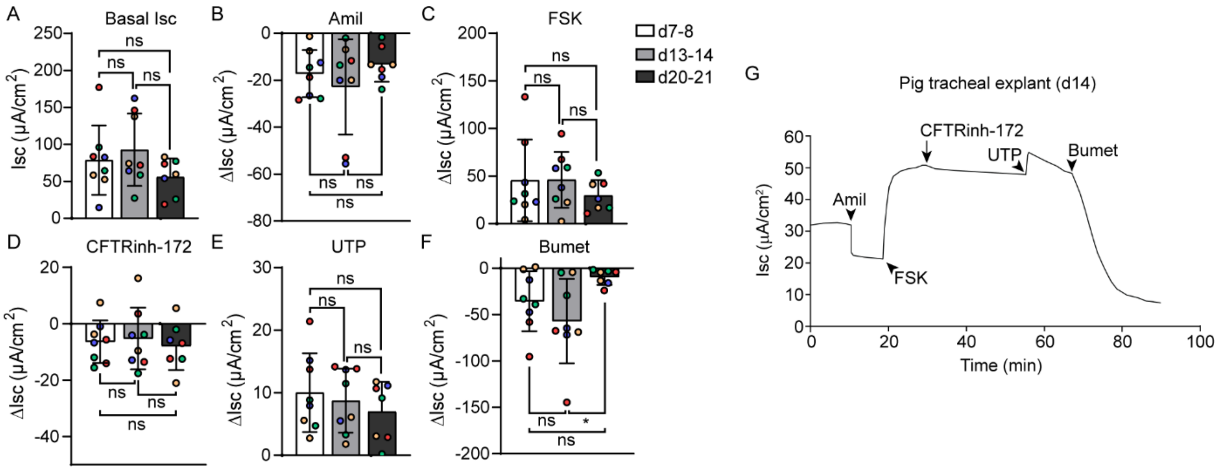

Fig. S5. Pig tracheal explant electrophysiology. (**A-F**) Time course of pig tracheal explant electrophysiology at d7-8, d13-14, and d20-21. (**A**) Basal Isc and ΔIsc in response to (**B**) Amil, (**C**) FSK, (**D**) CFTRinh-172, (**E**) UTP, and (**F**) Bumet. (**A-F**) N = 4 animals (represented by different colored dots); 1-2 replicates per animal. One-way ANOVA with Tukey's post-test; * = $p<0.05$. (**G**) Representative Ussing tracing of a d14 pig tracheal explant.

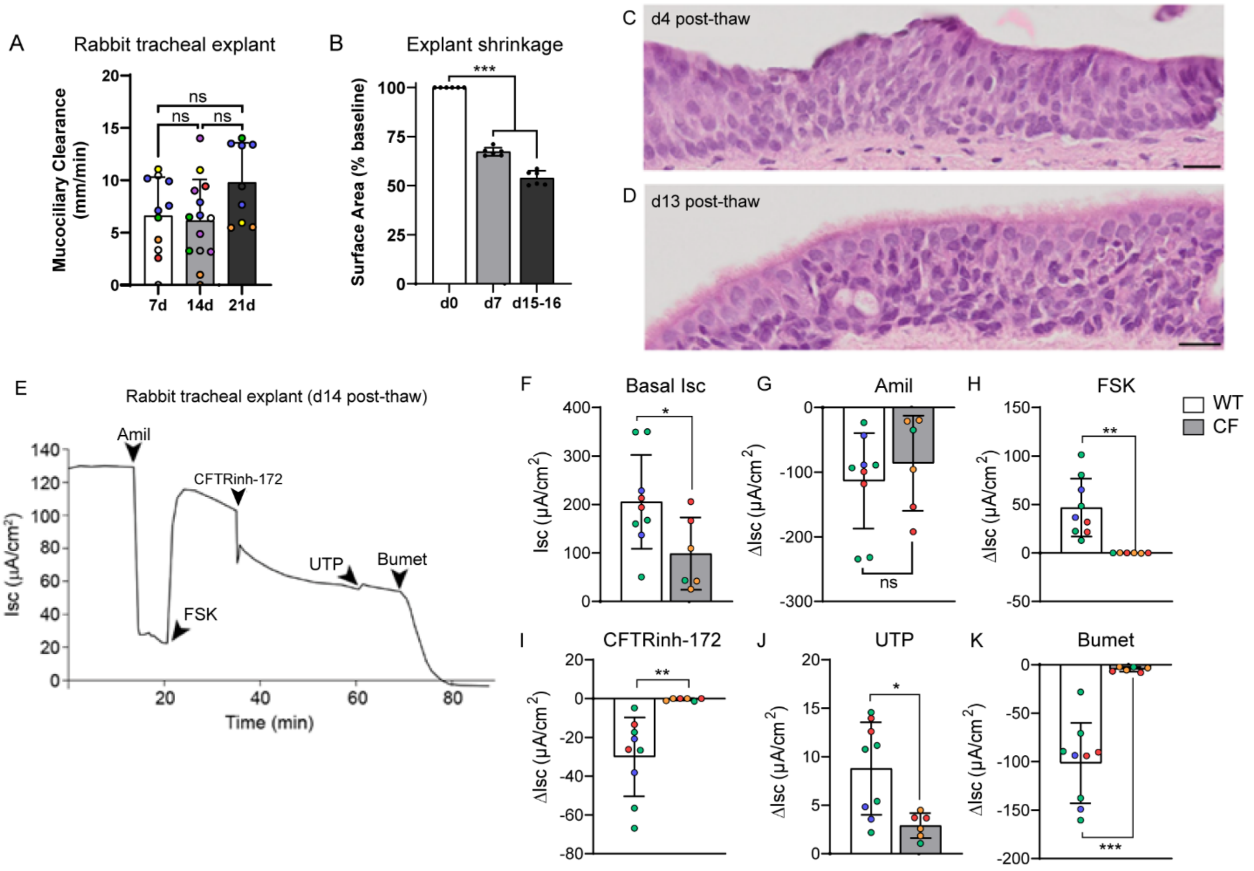

Fig. S6. Rabbit tracheal explant mucociliary clearance and cryopreservation data. (**A**) Mucociliary clearance of fluorescently labeled beads across rabbit tracheal explants after 7, 14, and 21 days in culture. N = 5-8 animals (represented by different colored dots); 1-4 replicates per animal. (**B**) Surface area of rabbit tracheal explants over time as a percent of baseline. N=1 animal; 6 replicates per animal. **(A-B)** One-way analysis of variance (ANOVA) with Tukey's post-test. (**C-D**) H&E histology of cryopreserved rabbit tracheal explants at 4 (**C**) and 13 (**D**) days post-thaw. Representative of N=3 animals. Scale bars = 20 µm. (**E-K**) Electrophysiology of cryopreserved wildtype (WT) versus CF rabbit tracheal explants at d7 post-thaw. (**E**) Representative Ussing tracing of a cryopreserved rabbit tracheal explant at d14 post-thaw. (**F**) Basal Isc and ΔIsc in response to (**G**) Amil, (**H**) FSK, (**I**) CFTRinh-172, (**J**) UTP, and (**K**) Bumet. (**D-J**) N=3 animals; 1-3 replicates per animal. (**F-K**) Unpaired T-test. ns = non-significant; * = p<0.05, ** = p<0.01, *** = p<0.001.

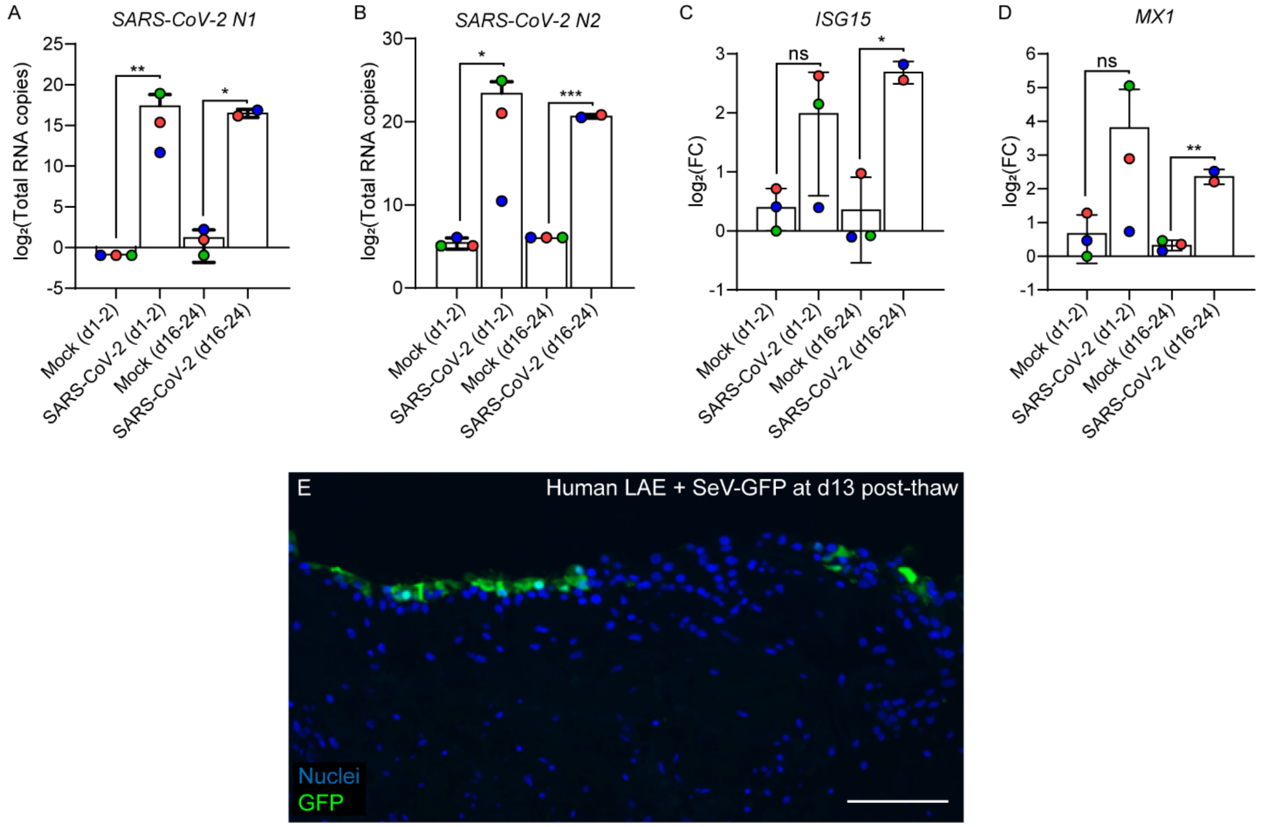

Fig. S7. SARS-CoV-2 infection of fresh versus cultured airway explants and cryopreserved tissues. (**A-B**) Log base 2 of the relative copy number of SARS-CoV-2 nucleocapsid genes *N1* (**A**) and *N2* (**B**) measured by qRT-PCR and compared to a standard curve in human LAE explants inoculated after 1-2 days of culture or 16-24 days of culture. Samples collected at 3 dpi. N = 2-3 donors; 1 replicate per donor. (**C-D**) Log base 2 of the relative gene expression of interferon stimulated genes *ISG15* (**C**) and *MX1* (**D**) measured by qRT-PCR in human LAE explants inoculated after 1-2 days of culture or 16-24 days of culture. Samples collected at 3 dpi. The fold change (FC) in gene expression was normalized to *TBP* expression. N = 2-3 donors; 1 replicate per donor. (**A-D**) Data were log transformed before statistical testing due to unequal variances amongst samples and analyzed using a linear mixed-effects model with the donor as a random effect factor; ns = non-significant; * = $p<0.05$, ** = $p<0.01$, *** = $p<0.001$. (**E**) Immunofluorescence of a human LAE made from cryopreserved airway tissue at 3 days post infection (dpi) following SeV-GFP inoculation at d13 post-thaw. Representative of N=3 donors. Scale bar = 100 μm.

Table S1. Cell cluster differential abundance testing. Cell abundance was compared across time points by performing a quasi-likelihood (QL) F-test on a sample-wise cell count matrix with a generalized linear model (GLM) fit and a negative binomial (NB) distribution. Values reported below include the log fold change (FC), log counts per million (CPM), F statistic (F), P-value, and false discovery rate (FDR). Significant FDRs are reported in Fig. 5.

Cluster ID	Model	Comparison	logFC	logCPM	F	P-Value	FDR
9. Suprabasal	LAE	d0 vs d2	6.842715	16.98433	17.72515	5.94E-05	0.001365
2. Basal	LAE	d0 vs d2	-5.51332	16.66587	13.46181	0.000408	0.004689
4. Suprabasal	LAE	d0 vs d2	5.237848	16.9554	11.5939	0.000982	0.00753
14. Alveolar fibroblast 2	LAE	d0 vs d2	-7.29853	13.50461	8.900873	0.003649	0.020984
15. Multiciliated cell	LAE	d0 vs d2	-3.25028	15.33339	5.408087	0.022242	0.102313
11. Proliferating Basal	LAE	d0 vs d2	2.522038	16.62083	3.768917	0.055274	0.211882
29. B cell	LAE	d0 vs d2	-4.64886	11.24949	3.264781	0.074051	0.239828
13. Multiciliated cell	LAE	d0 vs d2	-2.31131	15.25381	2.94006	0.089774	0.239828
7. Alveolar fibroblast 1	LAE	d0 vs d2	2.963227	13.44486	2.866134	0.093846	0.239828
24. Plasma cell	LAE	d0 vs d2	-3.08719	12.78331	2.040765	0.156519	0.331603
19. EC venous systemic	LAE	d0 vs d2	-2.04001	15.27469	2.020268	0.158593	0.331603
12. SMG duct	LAE	d0 vs d2	1.7301	16.3572	1.839404	0.17834	0.340108
5. Secretory	LAE	d0 vs d2	1.478786	16.85404	1.637512	0.203885	0.340108
16. AT2	LAE	d0 vs d2	2.402259	12.27775	1.614798	0.207023	0.340108
28. Mast cell	LAE	d0 vs d2	-1.98326	11.8672	1.180743	0.280044	0.4294
8. Pericyte	LAE	d0 vs d2	-1.50955	14.07012	1.012365	0.316976	0.455653
10. Macrophage	LAE	d0 vs d2	-1.18122	14.74314	0.730717	0.394871	0.534238
18. Myofibroblast	LAE	d0 vs d2	-0.97646	13.094	0.34443	0.558721	0.713921
0. T cell	LAE	d0 vs d2	-0.61297	16.23897	0.264216	0.608471	0.730986
30. PNECs & Ionocytes	LAE	d0 vs d2	-0.75545	12.16897	0.22599	0.63564	0.730986
1. EC general capillary	LAE	d0 vs d2	0.611688	13.0701	0.150553	0.698904	0.765466
20. Suprabasal	LAE	d0 vs d2	0.327244	15.26449	0.062786	0.802705	0.839191
27. Lymphatic EC	LAE	d0 vs d2	0.120501	11.86671	0.006096	0.937936	0.937936
4. Suprabasal	LAE	d0 vs d14	7.915907	16.9554	25.9573	1.85E-06	4.26E-05
2. Basal	LAE	d0 vs d14	-8.06028	16.66587	23.23606	5.64E-06	6.48E-05
9. Suprabasal	LAE	d0 vs d14	6.966136	16.98433	18.62232	4.01E-05	0.000307
14. Alveolar fibroblast 2	LAE	d0 vs d14	-9.46747	13.50461	11.52434	0.001015	0.005838
19. EC venous systemic	LAE	d0 vs d14	-5.04406	15.27469	9.390622	0.002862	0.013164
15. Multiciliated cell	LAE	d0 vs d14	-4.04412	15.33339	7.536872	0.00727	0.027868
13. Multiciliated cell	LAE	d0 vs d14	-3.69431	15.25381	6.159805	0.014883	0.048903
11. Proliferating Basal	LAE	d0 vs d14	-3.04265	16.62083	4.978791	0.028089	0.080757
10. Macrophage	LAE	d0 vs d14	-3.25299	14.74314	4.468356	0.037234	0.095153
7. Alveolar fibroblast 1	LAE	d0 vs d14	3.260763	13.44486	3.552653	0.062607	0.134497
24. Plasma cell	LAE	d0 vs d14	-3.92723	12.78331	3.505957	0.064325	0.134497
5. Secretory	LAE	d0 vs d14	1.870824	16.85404	2.636387	0.107862	0.206736
28. Mast cell	LAE	d0 vs d14	-2.69612	11.8672	2.225937	0.139131	0.246156
30. PNECs & Ionocytes	LAE	d0 vs d14	-1.69108	12.16897	1.028859	0.313087	0.514357
1. EC general capillary	LAE	d0 vs d14	-1.49843	13.0701	0.87963	0.350759	0.53783
18. Myofibroblast	LAE	d0 vs d14	-1.35656	13.094	0.728846	0.395475	0.568495
29. B cell	LAE	d0 vs d14	-1.52703	11.24949	0.612322	0.435924	0.58978
27. Lymphatic EC	LAE	d0 vs d14	-1.21599	11.86671	0.415212	0.520939	0.64203

20. Suprabasal	LAE	d0 vs d14	0.803085	15.26449	0.396674	0.530373	0.64203
12. SMG duct	LAE	d0 vs d14	0.87878	16.3572	0.336929	0.563027	0.647481
0. T cell	LAE	d0 vs d14	-0.6102	16.23897	0.249154	0.618863	0.677802
16. AT2	LAE	d0 vs d14	-0.65772	12.27775	0.093968	0.759885	0.794425
8. Pericyte	LAE	d0 vs d14	-0.31612	14.07012	0.039269	0.843354	0.843354
11. Proliferating Basal	LAE	d2 vs d14	-5.56469	16.62083	14.95096	0.000206	0.004728
19. EC venous systemic	LAE	d2 vs d14	-3.00406	15.27469	4.534705	0.035884	0.364786
4. Suprabasal	LAE	d2 vs d14	2.678059	16.9554	4.031891	0.047581	0.364786
2. Basal	LAE	d2 vs d14	-2.54696	16.66587	2.819728	0.096506	0.468382
16. AT2	LAE	d2 vs d14	-3.05997	12.27775	2.73107	0.101822	0.468382
10. Macrophage	LAE	d2 vs d14	-2.07177	14.74314	2.10955	0.149784	0.574174
1. EC general capillary	LAE	d2 vs d14	-2.11011	13.0701	1.825475	0.179976	0.591351
29. B cell	LAE	d2 vs d14	3.121826	11.24949	1.2323	0.269853	0.767461
13. Multiciliated cell	LAE	d2 vs d14	-1.383	15.25381	1.085002	0.300311	0.767461
8. Pericyte	LAE	d2 vs d14	1.193422	14.07012	0.657379	0.419579	0.879995
12. SMG duct	LAE	d2 vs d14	-0.85132	16.3572	0.42926	0.513987	0.879995
27. Lymphatic EC	LAE	d2 vs d14	-1.33649	11.86671	0.416204	0.520443	0.879995
15. Multiciliated cell	LAE	d2 vs d14	-0.79384	15.33339	0.356507	0.551919	0.879995
14. Alveolar fibroblast 2	LAE	d2 vs d14	-2.16894	13.50461	0.31141	0.578172	0.879995
30. PNECs & Ionocytes	LAE	d2 vs d14	-0.93563	12.16897	0.294175	0.588869	0.879995
24. Plasma cell	LAE	d2 vs d14	-0.84004	12.78331	0.198398	0.657063	0.879995
28. Mast cell	LAE	d2 vs d14	-0.71285	11.8672	0.131138	0.718085	0.879995
20. Suprabasal	LAE	d2 vs d14	0.47584	15.26449	0.129931	0.719329	0.879995
5. Secretory	LAE	d2 vs d14	0.392038	16.85404	0.122674	0.726953	0.879995
18. Myofibroblast	LAE	d2 vs d14	-0.3801	13.094	0.056405	0.812799	0.929041
7. Fibroblast	LAE	d2 vs d14	0.297536	13.44486	0.03682	0.848255	0.929041
9. Suprabasal	LAE	d2 vs d14	0.123421	16.98433	0.011983	0.913072	0.954575
0. T cell	LAE	d2 vs d14	0.002769	16.23897	5.81E-06	0.998083	0.998083
21. Classical monocyte	SAE	d0 vs d2	-10.8527	14.92288	41.33616	3.58E-09	9.65E-08
7. Alveolar fibroblast 1	SAE	d0 vs d2	7.729709	16.1714	32.01139	1.27E-07	1.38E-06
2. Basal	SAE	d0 vs d2	-6.96293	15.40887	31.53668	1.54E-07	1.38E-06
4. Suprabasal	SAE	d0 vs d2	6.314819	15.82699	23.57998	4.07E-06	2.75E-05
14. Alveolar fibroblast 2	SAE	d0 vs d2	-3.81435	14.50414	15.53708	0.000144	0.000659
9. Suprabasal	SAE	d0 vs d2	5.002419	14.96498	15.49965	0.000146	0.000659
26. Pericyte	SAE	d0 vs d2	-3.39447	12.6233	13.80566	0.000323	0.001246
12. SMG duct	SAE	d0 vs d2	3.054169	13.28541	9.762667	0.002287	0.007719
11. Proliferating Basal	SAE	d0 vs d2	2.287599	13.61649	7.317045	0.007938	0.023815
20. Suprabasal	SAE	d0 vs d2	2.476791	14.85203	6.117187	0.014946	0.040353
1. EC general capillary	SAE	d0 vs d2	2.255983	17.0782	5.478856	0.021084	0.051752
15. Multiciliated cell	SAE	d0 vs d2	-2.1179	15.01086	5.141689	0.025348	0.057034
16. AT2	SAE	d0 vs d2	-2.20869	15.62333	4.860423	0.0296	0.060896
8. Pericyte	SAE	d0 vs d2	2.084599	16.00347	4.743999	0.031576	0.060896
10. Macrophage	SAE	d0 vs d2	-1.99162	15.8312	4.347856	0.039413	0.070943
25. AT1	SAE	d0 vs d2	-1.18139	14.1086	2.027062	0.157402	0.265615
18. Myofibroblast	SAE	d0 vs d2	1.162196	14.73405	1.805696	0.181842	0.288807
24. Plasma cell	SAE	d0 vs d2	-0.72711	13.56082	0.786927	0.377002	0.565503
30. PNECs & Ionocytes	SAE	d0 vs d2	-0.85874	11.00721	0.569034	0.452285	0.642721

28. Mast cell	SAE	d0 vs d2	0.516081	12.49868	0.405325	0.525699	0.696904
5. Secretory	SAE	d0 vs d2	0.62624	15.7496	0.374149	0.542037	0.696904
19. EC venous systemic	SAE	d0 vs d2	-0.27761	14.51962	0.108225	0.742811	0.888735
27. Lymphatic EC	SAE	d0 vs d2	-0.23966	13.63996	0.090916	0.763596	0.888735
17. EC aerocyte capillary	SAE	d0 vs d2	0.194978	15.71267	0.046942	0.82888	0.888735
0. T cell	SAE	d0 vs d2	-0.19621	16.69466	0.046246	0.830135	0.888735
13. Multiciliated cell	SAE	d0 vs d2	0.156968	14.62841	0.033173	0.855819	0.888735
29. B cell	SAE	d0 vs d2	-0.02926	12.97941	0.001303	0.971275	0.971275
2. Basal	SAE	d0 vs d14	-11.4895	15.40887	45.27881	8.49E-10	2.29E-08
21. Classical monocyte	SAE	d0 vs d14	-11.1417	14.92288	42.19705	2.60E-09	3.51E-08
7. Alveolar fibroblast 2	SAE	d0 vs d14	8.828565	16.1714	40.69205	4.54E-09	3.58E-08
14. Alveolar fibroblast 1	SAE	d0 vs d14	-8.80524	14.50414	40.27273	5.31E-09	3.58E-08
4. Suprabasal	SAE	d0 vs d14	8.166106	15.82699	37.74828	1.37E-08	7.41E-08
26. Pericyte	SAE	d0 vs d14	-6.9499	12.6233	30.93075	1.96E-07	8.80E-07
9. Suprabasal	SAE	d0 vs d14	7.033601	14.96498	30.06834	2.77E-07	1.07E-06
25. AT1	SAE	d0 vs d14	-4.28028	14.1086	19.18267	2.76E-05	9.31E-05
8. Pericyte	SAE	d0 vs d14	4.29551	16.00347	16.72692	8.34E-05	0.00025
10. Macrophage	SAE	d0 vs d14	-3.83744	15.8312	14.54038	0.000229	0.000618
15. Multiciliated cell	SAE	d0 vs d14	-3.41347	15.01086	11.77627	0.000851	0.002089
5. Secretory	SAE	d0 vs d14	2.591693	15.7496	7.508174	0.007188	0.016173
12. SMG duct	SAE	d0 vs d14	2.008459	13.28541	4.807879	0.030475	0.058263
19. EC venous systemic	SAE	d0 vs d14	-1.92387	14.51962	4.724225	0.031925	0.058263
13. Multiciliated cell	SAE	d0 vs d14	-1.93515	14.62841	4.699439	0.032368	0.058263
29. B cell	SAE	d0 vs d14	1.339172	12.97941	2.767192	0.099114	0.167255
20. Suprabasal	SAE	d0 vs d14	1.32271	14.85203	1.970544	0.163259	0.259294
11. Basal	SAE	d0 vs d14	-1.23191	13.61649	1.610723	0.207118	0.310678
16. AT2	SAE	d0 vs d14	-1.1964	15.62333	1.392866	0.240515	0.341784
30. PNECs & Ionocytes	SAE	d0 vs d14	0.900538	11.00721	0.838208	0.361949	0.488632
24. Plasma cell	SAE	d0 vs d14	-0.68356	13.56082	0.743815	0.390353	0.501882
18. Myofibroblast	SAE	d0 vs d14	-0.73557	14.73405	0.680667	0.411176	0.504625
28. Mast cell	SAE	d0 vs d14	0.527672	12.49868	0.420462	0.518083	0.594156
17. EC aerocyte capillary	SAE	d0 vs d14	-0.58847	15.71267	0.400559	0.528138	0.594156
0. T cell	SAE	d0 vs d14	-0.08121	16.69466	0.008093	0.928485	0.979328
1. EC general capillary	SAE	d0 vs d14	0.071889	17.0782	0.005126	0.943056	0.979328
27. Lymphatic EC	SAE	d0 vs d14	0.012539	13.63996	0.000206	0.988567	0.988567
11. Proliferating basal	SAE	d2 vs d14	-3.51951	13.61649	13.82946	0.000319	0.008622
14. Alveolar fibroblast 2	SAE	d2 vs d14	-4.99088	14.50414	11.61196	0.000922	0.012443
25. AT1	SAE	d2 vs d14	-3.09889	14.1086	9.623026	0.002452	0.02207
26. Pericyte	SAE	d2 vs d14	-3.55544	12.6233	6.053564	0.015463	0.101128
8.Pericyte	SAE	d2 vs d14	2.21091	16.00347	5.212822	0.024378	0.101128
1. EC general capillary	SAE	d2 vs d14	-2.18409	17.0782	5.151315	0.025215	0.101128
13. Multiciliated cell	SAE	d2 vs d14	-2.09212	14.62841	5.033616	0.0269	0.101128
9. Suprabasal	SAE	d2 vs d14	2.031182	14.96498	4.733632	0.031758	0.101128
18. Myofibroblast	SAE	d2 vs d14	-1.89776	14.73405	4.626601	0.033709	0.101128
5. Secretory	SAE	d2 vs d14	1.965453	15.7496	4.141456	0.044294	0.119594
2. Basal	SAE	d2 vs d14	-4.52656	15.40887	3.971261	0.048806	0.119795
4. Suprabasal	SAE	d2 vs d14	1.851287	15.82699	3.803939	0.053724	0.120878

19. EC venous systemic	SAE	d2 vs d14	-1.64626	14.51962	3.523235	0.063213	0.130808
10. Macrophage	SAE	d2 vs d14	-1.84582	15.8312	3.402797	0.067826	0.130808
29. B cell	SAE	d2 vs d14	1.368434	12.97941	2.852485	0.094118	0.163206
30. PNECs & Ionocytes	SAE	d2 vs d14	1.759279	11.00721	2.807545	0.096715	0.163206
15. Multiciliated cell	SAE	d2 vs d14	-1.29558	15.01086	1.578054	0.211752	0.336312
12. SMG duct	SAE	d2 vs d14	-1.04571	13.28541	1.40936	0.237767	0.339876
20. Suprabasal	SAE	d2 vs d14	-1.15408	14.85203	1.400897	0.239172	0.339876
7. Alveolar fibroblast 1	SAE	d2 vs d14	1.098856	16.1714	1.303184	0.256157	0.345812
16. AT2	SAE	d2 vs d14	1.012289	15.62333	1.085742	0.299743	0.385384
17. EC aerocyte capillary	SAE	d2 vs d14	-0.78345	15.71267	0.732622	0.39393	0.483459
27. Lymphatic EC	SAE	d2 vs d14	0.252196	13.63996	0.097142	0.755888	0.887347
0. T cell	SAE	d2 vs d14	0.115006	16.69466	0.015933	0.899789	0.999991
24. Plasma cell	SAE	d2 vs d14	0.043548	13.56082	0.003154	0.955318	0.999991
28. Mast cell	SAE	d2 vs d14	0.011592	12.49868	0.000229	0.987944	0.999991
21. Classical monocyte	SAE	d2 vs d14	-0.28894	14.92288	1.23E-10	0.999991	0.999991
7. Alveolar fibroblast 1	PCLS	d0 vs d14	7.54909	16.73601	57.00379	0.000127	0.00194
26. Pericyte	PCLS	d0 vs d14	-7.13066	14.69778	71.86444	0.000169	0.00194
19. EC venous systemic	PCLS	d0 vs d14	-3.39997	13.6213	39.9647	0.000408	0.003126
25. AT1	PCLS	d0 vs d14	-2.93357	14.70155	36.79058	0.001209	0.006954
8. Pericyte	PCLS	d0 vs d14	3.817626	16.64794	32.13524	0.001944	0.008945
17. EC aerocyte capillary	PCLS	d0 vs d14	-2.77998	15.40233	26.18038	0.0033	0.012649
14. Alveolar fibroblast 2	PCLS	d0 vs d14	-7.4222	16.78638	17.85263	0.003984	0.013089
12. SMG duct	PCLS	d0 vs d14	5.266683	13.51616	21.89751	0.005774	0.0166
11. Proliferating basal	PCLS	d0 vs d14	4.712372	12.60392	12.10971	0.01257	0.032124
1. EC general capillary	PCLS	d0 vs d14	-2.12226	16.82341	7.169783	0.042571	0.097914
21. Classical monocyte	PCLS	d0 vs d14	-4.09151	14.68313	3.910606	0.091992	0.185013
5. Secretory	PCLS	d0 vs d14	4.523002	12.66963	4.542498	0.100951	0.185013
16. AT2	PCLS	d0 vs d14	1.225161	15.57584	3.871076	0.104573	0.185013
28. Mast cell	PCLS	d0 vs d14	0.950187	13.6799	3.053604	0.134732	0.221345
4. Suprabasal	PCLS	d0 vs d14	4.32813	12.01751	2.886285	0.170714	0.261762
18. Myofibroblast	PCLS	d0 vs d14	1.185596	16.16305	2.164029	0.199589	0.2665
20. Suprabasal	PCLS	d0 vs d14	1.880825	12.74574	1.899594	0.20839	0.2665
13. Multiciliated cell	PCLS	d0 vs d14	3.161161	15.61893	2.206354	0.208565	0.2665
0. T cell	PCLS	d0 vs d14	-0.82884	16.5879	1.917902	0.223108	0.270078
29. B cell	PCLS	d0 vs d14	-1.06505	12.68067	1.011234	0.350278	0.40282
24. Plasma cell	PCLS	d0 vs d14	0.298735	14.75977	0.112106	0.749113	0.820457
27. Lymphatic EC	PCLS	d0 vs d14	0.154796	12.80136	0.043555	0.841448	0.874356
10. Macrophage	PCLS	d0 vs d14	0.089744	16.24179	0.027611	0.874356	0.874356

Table S2. Summary of statistically significant preserved properties in airway explant models.

Electrophysiology								
**Tissue**	**Comparison**	**Basal Isc**	**Amil**	**FSK**	**CFTRinh-172**	**UTP**	**Bumet**	**Conclusion**
**LAE**	d1-2 vs d14	Decrease	Decrease	Increase	No change	Decrease	ns	LAE and SAE are injured by dissection and cold ischemia but recover with time in culture
**SAE**	d1-2 vs d14	ns	ns	Increase	Increase	Decrease	ns	
**LAE**	d14 vs d14 post-thaw	ns	ns	ns	ns	ns	ns	Explants regenerate by d14 post-thaw

Cell proportion changes					
**Tissue**	**Comparison**	**Decreasing**	**Increasing**	**Transient Increase**	**Conclusion**
**LAE**	d0 vs d2 vs d14	Basal cells, multiciliated cells, venous systemic ECs	Suprabasal cells	Proliferating basal cells	Diverse cell lineages are preserved in the explant models, however quiescent cell populations lost over time and replaced with activated and/or repairing cell subsets
**SAE**	d0 vs d2 vs d14	Basal cells, multiciliated cells, AT1 cells, macrophages, classical monocytes, a subset of alveolar fibroblasts (cluster 14), and a subset of pericytes (cluster 26)	Suprabasal cells, secretory cells, a subset of alveolar fibroblast (cluster 7), and a subset of pericytes (cluster 8)	Proliferating basal cells	
**PCLS**	d0 vs d2 vs d14	AT1, venous systemic ECs, aerocyte capillary ECs, a subset of alveolar fibroblasts (cluster 14), and a subset of pericytes (cluster 26)	A subset of alveolar fibroblast (cluster 7) and a subset	-	

Viral Infection and ISG response				
**Tissue**	**Comparison**	**SARS-CoV-2 N1 and N2**	**ISG response**	**Conclusion**
**LAE**	d1-2 vs d16-24	ns	ns	LAE infection and response to infection is preserved across time in explant cultures

Table S3. Formalin fixed paraffin embedded (FFPE) immunohistochemistry protocol. Primary and secondary antibodies along with antibody source, catalog number, concentration, and Research Resource Identifiers (RRID).

**Primary Antibody Target**	**Source**	**Catalog number**	**Concentration**	**RRID**
a-Tubulin	Millipore	MAB1864	3 µg/ml	RRID:AB_2210391
MUC5B	Sigma-Aldrich	HPA008246	0.1 µg/ml	RRID:AB_1854203
MUC5AC	Thermo Fisher Scientific	MA5-12178	4 µg/ml	RRID:AB_10978001
PECAM1	AbCam	ab76533	1.3 µg/ml	RRID:AB_1523298
ACTA2	LSBio (LifeSpan)	B3933	1 µg/ml	RRID:AB_10686658
PTPRC	Thermo Fisher Scientific	14-0459-82	1 µg/ml	RRID:AB_467274
CD68	Cell Signaling Technology	76437	1.5 µg/ml	RRID:AB_2799882
CD3	AbCam	ab16669	1:100	RRID:AB_443425
CD20	Agilent	M0755	1:200	RRID:AB_2282030
RAGE/AGER	R&D Systems	AF1145	0.4 µg/ml	RRID:AB_354628
Pro-SPB	Seven Hills Bioreagents	WRAB-55522	1:1000	RRID:AB_2938816
LAMP-3	Novus	DDX0191P-100	5 µg/ml	RRID:AB_2827532
Ki-67	BD Biosciences	550609	1 µg/ml	RRID:AB_393778
PDGFRA	R&D Systems	AF-307-NA	1:300	RRID:AB_354459
Keratin 5	Biolegend	905901	2 µg/ml	RRID:AB_2565054
GFP	AbCam	ab6556	0.5 µg/ml	RRID:AB_305564
SARS-CoV-2 N	Thermo Fisher Scientific	PA1-41098	1 µg/ml	RRID:AB_1087200
**Secondary Antibodies**	**Source**	**Catalog number**	**Concentration**	**RRID**
Donkey anti-mouse 488	Jackson ImmunoResearch Labs	715-545-151	3.125 µg/ml	RRID:AB_2341099
Donkey anti-rabbit 488	Jackson ImmunoResearch Labs	711-545-152	3.125 µg/ml	RRID:AB_2313584
Donkey anti-goat 488	Jackson ImmunoResearch Labs	705-545-147	3.125 µg/ml	RRID:AB_2336933
Donkey anti-rabbit 555	Thermo Fisher Scientific	A31572	2 µg/ml	RRID:AB_162543
Donkey anti-rabbit 594	Jackson ImmunoResearch Labs	711-585-152	3.125 µg/ml	RRID:AB_2340621
Donkey anti-rat 594	Thermo Fisher Scientific	A21209	2 µg/ml	RRID:AB_2535795
Donkey anti-mouse 647	Thermo Fisher Scientific	A31571	2 µg/ml	RRID:AB_162542
Donkey anti-goat 647	Thermo Fisher Scientific	A21447	2 µg/ml	RRID:AB_2535864
Donkey anti-chicken 647	Jackson ImmunoResearch Labs	703-605-155	3.125 µg/ml	RRID:AB_2340379

Table S4. FFPE RNA in situ hybridization protocol. RNA in situ hybridization probes along with probe source, and catalog number.

**Colorimetric RNA in situ hybridization probe**	**Source**	**Catalog number**
RNAscope™ Probe- Hs-FOXJ1	Advanced Cell Diagnostics, Inc.	430921
RNAscope™ Probe- Hs-SCGB1A1	Advanced Cell Diagnostics, Inc.	469971
RNAscope™ Probe- Hs-KRT5-O1	Advanced Cell Diagnostics, Inc.	547901
RNAscope™ Probe- Hs-CFTR	Advanced Cell Diagnostics, Inc.	603291
RNAscope™ Probe- Hs-CD68	Advanced Cell Diagnostics, Inc.	560591
RNAscope™ Probe- Hs-SFTPB	Advanced Cell Diagnostics, Inc.	544251
RNAscope™ Probe- Hs-AGER	Advanced Cell Diagnostics, Inc.	470121
**Fluorescent RNA in situ hybridization probes**		
RNAscope™ Probe- Hs-SCGB1A1	Advanced Cell Diagnostics, Inc.	469971
RNAscope™ Probe- Hs-FOXJ1-C2	Advanced Cell Diagnostics, Inc.	430921-C2
RNAscope™ Probe- V-nCoV2019-S-C3	Advanced Cell Diagnostics, Inc.	848561-C3
RNAscope™ Probe- Hs-SFTPC	Advanced Cell Diagnostics, Inc.	452561
RNAscope™ Probe- Hs-CFTR-C2	Advanced Cell Diagnostics, Inc.	603291-C2
RNAscope™ Probe- Hs-SCGB1A1-C3	Advanced Cell Diagnostics, Inc.	469971-C3

Table S5. Raw data for the WST-8 viability assay reported in Fig. 2C. Serial measurements were performed over time and data was normalized to the d1 measurement for each PCLS.

Donor ID	Condition	d1	d4	d14	d21	d28	d36	d42
DD052S	Submerged	0.7198	0.6033	0.2506	0.4094	0.3861	0.3458	0.2127
DD052S	Submerged	0.9145	0.6762	0.3763	0.6437	0.6103	0.592	0.4529
DD052S	Submerged	0.7424	0.6054	0.5417	0.7741	0.6838	0.5493	0.3597
DD001T	Submerged	2.0581	2.3172	2.0722	1.6011	1.2349	1.255	-
DD001T	Submerged	1.8347	2.5458	1.7292	1.7360	1.6141	1.5127	1.05605
DD001T	Submerged	2.4251	2.5694	1.9277	2.4049	2.0605	1.9365	1.54715
DD004T	Submerged	1.4152	1.4458	2.0123	1.8996	1.60415	1.30475	0.9534
DD004T	Submerged	1.6231	1.4641	1.9732	0.8774	1.01205	0.88225	0.5492
DD004T	Submerged	1.4018	1.3943	1.701	1.3537	1.05225	1.31865	1.6201
DD052S	Gelfoam	0.6673	0.601	0.5826	0.5769	0.6291	0.7323	0.6459
DD052S	Gelfoam	0.6537	0.5617	0.3078	0.5271	0.5418	0.5889	0.5591
DD052S	Gelfoam	0.4924	0.3871	0.3871	0.3777	0.4381	0.5121	0.2512
DD001T	Gelfoam	2.226	2.2283	2.1928	2.3087	1.7263	1.2791	1.28535
DD001T	Gelfoam	2.2461	2.3564	2.5024	1.7173	1.1427	1.1727	0.94845
DD001T	Gelfoam	2.4537	2.3362	2.647	2.5663	1.8411	2.0232	1.52055
DD004T	Gelfoam	1.3822	1.5461	1.9884	2.0264	1.13205	1.48365	1.8386
DD004T	Gelfoam	1.4362	1.3925	1.7861	1.4407	1.02595	0.90185	0.8402
DD004T	Gelfoam	1.4788	1.6936	1.8582	2.0275	1.54275	1.67305	1.5947

Table S6. Raw data for LAE Ussing measurements reported in Fig. 3, A to F. Baseline short circuit current (Isc) reported in μA/cm^2, baseline resistance (R) reported in Ω/cm^2, baseline potential difference (PD) reported in mV. All other measurements are changes in Isc reported in μA/cm^2.

Ussing Date	Donor ID	Airway Size	Time in Culture	Isc	R	PD	Amil	FSK	CFTRinh-172	UTP	Bumet
9.16.20	DD057Q	LAE	24h	29.21	37.4	1.09	-10.26	1.99	-0.9	1.93	-0.48
1.13.21	DD001R	LAE	48h	89.06	104.6	9.32	-59.75	13.58	-3.24	10.9	-26.93
1.13.21	DD001R	LAE	48h	61.86	61.32	3.79	-36.32	11.69	0.06	1.47	-14.93
3.10.21	DD008R	LAE	48h	85.34	104.6	8.93	-53.73	6.86	-9.86	15.49	-10.61
3.10.21	DD008R	LAE	48h	60.58	35.86	2.17	-19.11	4.69	1.01	4.65	-4.31
4.29.21	DD017R	LAE	48h	172.9	68.26	11.8	-82.54	13.78	-4.43	12.54	-74.18
4.29.21	DD017R	LAE	48h	183.76	80.13	14.72	-84.1	7.7	-34.19	10.72	-45.42
6.09.21	DD023R	LAE	48h	130.02	59.05	7.68	-67.36	14.54	-11.6	23.25	-20.08
6.09.21	DD023R	LAE	48h	114.05	79.52	9.07	-75.06	10.1	-5.39	11.5	-3.33
9.30.20	DD057Q	LAE	14d	20.55	50.48	1.04	-9.92	7.95	-4.99	2.15	0
9.30.20	DD057Q	LAE	14d	42.75	38.92	1.66	-19.6	17.43	-16.13	3.81	0
1.25.21	DD001R	LAE	14d	49.42	69.58	3.44	-27.54	23.85	-3.33	1.3	-15.34
1.25.21	DD001R	LAE	14d	28.1	73.34	2.06	-17.43	15.38	-11.14	1.51	-5.46
3.23.21	DD008R	LAE	15d	66.57	46.71	3.11	-33.54	48.22	-59.7	7.18	-7.04
4.7.21	DD012R	LAE	15d	40.9	48.73	1.99	-19.98	24	-25.91	8.5	-9.34
4.7.21	DD012R	LAE	15d	32.21	74.69	2.41	-13.88	19.24	-17.74	5.44	-9.06
6.21.21	DD023R	LAE	14d	97.02	52.57	5.1	-50.7	63.3	-65.93	7.27	-14.02
6.21.21	DD023R	LAE	14d	37.27	71.71	2.67	-14.49	14.6	-12.16	4.67	-5.51

Table S7. Raw data for SAE Ussing measurements reported in Fig. 3, G to L. Baseline short circuit current (Isc) reported in µA/cm^2, baseline resistance (R) reported in Ω/cm^2, baseline potential difference (PD) reported in mV. All other measurements are changes in Isc reported in µA/cm^2.

**Ussing Date**	**Donor ID**	**Airway size**	**Time in Culture**	**Isc**	**R**	**PD**	**Amil**	**FSK**	**CFTRinh-172**	**UTP**	**Bumet**
8.27.20	DD054Q	SAE	24h	66.9	6.12	0.41	-37.6	25.15	-14.99	13.73	-37.81
3.25.21	DD012R	SAE	48h	14.53	34.43	0.5	-27.52	12.99	-3.24	16.62	0
1.13.21	DD001R	SAE	48h	57.63	96.75	5.58	-41.5	13.02	-5.29	5.61	-12.28
4.29.21	DD017R	SAE	48h	20.15	21.99	0.44	-2.29	3.92	-2.47	6.91	-0.48
4.29.21	DD017R	SAE	48h	28.47	56.82	1.62	-4.87	8.55	-1.62	14.42	-6.07
7.01.21	DD027R	SAE	48h	78.5	116.39	9.14	-39.86	7.94	-17.79	13.52	-4.74
11.05.20	DD065Q	SAE	48h	36.53	64.87	2.37	-15.85	6.21	-2.36	6.83	-8.06
11.05.20	DD065Q	SAE	48h	37.54	42.14	1.58	-15.74	2.34	-5.6	5.78	-10.22
9.9.20	DD054Q	SAE	14d	87.55	61.39	5.38	-37.56	39.5	-24.92	3.74	-36.31
4.7.21	DD012R	SAE	15d	61.67	35.12	2.17	-36.02	25.79	-18.16	8.78	-14.5
4.7.21	DD012R	SAE	15d	38.69	74.63	2.89	-24.91	22.8	-10.47	4.51	-9.64
1.25.21	DD001R	SAE	14d	68.36	48.47	3.31	-44.84	45.56	-18.7	3.98	-15.5
1.25.21	DD001R	SAE	14d	60.27	85.03	5.13	-51.72	27.7	-18.2	3.87	-8.73
5.11.21	DD017R	SAE	14d	114.84	37.72	4.33	-91.02	37.39	-59.89	5.32	-2.02
5.11.21	DD017R	SAE	14d	21.96	23.47	0.52	-20.29	8.28	-12.41	1.85	-2.05
7.13.21	DD027R	SAE	14d	90.94	73.1	6.65	-53.78	49.14	-51.3	6.02	-13.53
7.13.21	DD027R	SAE	14d	18.17	47.15	0.86	-20.71	19.71	-12.38	3.14	-8.9
2.08.21	DD004R	SAE	14d	32.71	32.65	1.07	-14.71	14.43	-11.29	6.65	-21.68
2.08.21	DD004R	SAE	14d	28.18	24.48	0.69	-10.52	18.85	-32.64	2.27	

Table S8. Raw data for CF versus non-CF Ussing measurements reported in Fig. 3, M to R. Baseline short circuit current (Isc) reported in μA/cm^2, baseline resistance (R) reported in Ω/cm^2, baseline potential difference (PD) reported in mV. All other measurements are changes in Isc reported in μA/cm^2.

Ussing Date	CF status	Donor ID	Airway size	Time in Culture	Isc	R	PD	Amil	FSK	CFTRinh-172	UTP	Bumet
10.21.20	CF	KKCFFT010Q	LAE	14d	49.84	37.81	1.88	-13.74	1.98	0	4.63	0.08
11.19.20	CF	KKCFFT011Q	LAE	13d	34.24	28.21	0.97	-14.14	1.76	-3.03	2.3	0
11.19.20	CF	KKCFFT011Q	LAE	13d	23.78	25.78	0.61	-4.12	0	0	-1.07	-1.52
2.04.21	CF	KKCFFT002R	LAE	14d	44.38	45.94	2.04	-15.41	0	-1.01	1.5	-1.6
2.04.21	CF	KKCFFT002R	LAE	14d	28.26	42.12	1.19	-8.77	0	2.13	1.31	0
2.19.21	CF	KKD003R	LAE	14d	26.04	72.05	1.88	-20.19	-0.22	-1.11	2.84	-0.97
2.25.21	CF	KKCFFT004R	LAE	14d	66.62	71.44	1	-51.71	-0.56	-0.62	1.22	-0.87
2.25.21	CF	KKCFFT004R	LAE	14d	27.85	49.98	1.31	-29.27	-0.78	-1.6	0.59	-0.28
10.4.21	CF	KKCFFT007R	LAE	14d	21.83	39.85	0.87	-21.83	0.38	-3.55	2.29	-1.74
10.4.21	CF	KKCFFT007R	LAE	14d	12.24	24.88	0.3	-13.88	-0.77	-1.36	3.03	-1
10.27.20	Non-CF	DD063Q	LAE	14d	53.48	37.92	2.03	-23.49	9.15	-4.68	2.55	-2.48
10.27.20	Non-CF	DD063Q	LAE	14d	34.28	40.27	1.38	-8.98	10.66	-5.83	5.39	0
9.9.20	Non-CF	DD054Q	LAE	14d	23.91	43.14	1.03	-2.51	9.67	-3.28	2.4	-23
2.08.21	Non-CF	DD004R	LAE	14d	32.53	30.96	1.01	-1.87	43.52	-16.89	3.87	-13.5
4.30.21	Non-CF	DD015R	LAE	14d	48.19	32.46	1.56	-2.36	12.48	-11.5	5.38	-15.07
5.11.21	Non-CF	DD017R	LAE	14d	54.78	63.29	3.47	-27.31	20.16	-25.18	9.46	-7.21

Table S9. Raw data for rabbit tracheal explant Ussing measurements reported in Fig. 6, G to L. Baseline short circuit current (Isc) reported in μA/cm^2, baseline resistance (R) reported in Ω/cm^2, baseline potential difference (PD) reported in mV. All other measurements are changes in Isc reported in μA/cm^2.

Ussing Date	Rabbit ID	Sex	Age of Rabbit	Days in culture	Isc	R	PD	Amil	FSK	CFTRinh-172	UTP	Bumet
10.7.20	RAB002Q	M	292d	7d	112.17	91.51	10.26	-78.43	51.96	-6.98	14.09	-62.77
10.7.20	RAB002Q	M	292d	7d	317.53	64	20.32	-137.45	51.32	-51.23	13.23	-135.87
10.27.20	RAB004Q	M	568d	7d	176.6	55.02	9.72	-157.26	67.47	-31.26	21.57	-80.36
11.16.20	RAB005Q	F	31d	7d	252.53	72.83	18.39	-156.02	47.51	-11.26	34.17	-137.06
1.12.21	RAB002R	M	433d	7d	100.2	54.43	5.44	-73.87	45.77	-10.22	9.81	-53.4
1.12.21	RAB002R	M	433d	7d	166.22	48.83	8.12	-119.08	86.46	-36.54	21.25	-95.32
3.10.21	RAB004R	M	26d	7d	280.57	45.69	12.82	-94.11	88.11	-20.26	33.21	-233.36
4.15.21	RAB005R	M	428d	7d	129.18	64.42	8.32	-108.51	69.3	-17.3	10.13	-60.97
4.15.21	RAB005R	M	428d	7d	260.17	40.96	10.66	-264.45	121.92	-120.38	17.27	-57.84
10.14.20	RAB002Q	M	292d	14d	116.37	102.71	11.95	-36.49	25.1	-10.2	8.46	-51.9
10.14.20	RAB002Q	M	292d	14d	102.88	161.73	16.64	-36.3	16.45	-7.84	5.21	-41.87
11.3.20	RAB004Q	M	568d	14d	98.11	114.55	11.24	-89.6	47.83	-22.13	11.38	-51.94
11.23.20	RAB005Q	F	31d	14d	221.85	61.42	13.63	-114.7	88.36	-10.26	23.98	-123.09
11.23.20	RAB005Q	F	31d	14d	193.3	52.49	10.15	-104.15	87.49	-10.4	15.04	-103.7
4.22.21	RAB005R	M	428d	14d	125.36	41.12	5.16	-95.18	68.22	-24.79	14.56	-37.66
4.22.21	RAB005R	M	428d	14d	120.82	47.056	5.69	-133.2	84.82	-24.54	12.94	-75.92
10.21.21	RAB002Q	M	292d	21d	109.14	150.27	16.4	-51.57	11.39	-24.6	5.23	-1.61
11.10.20	RAB004Q	M	568d	21d	106.55	128	13.64	-39.56	54.61	-16.44	13.5	-96.73
2.04.21	RAB002R	M	433d	21d	396.79	28.7	11.39	-87.32	5.89	-63.96	4.89	-95.85
2.04.21	RAB002R	M	433d	21d	104.64	62.24	6.51	-69.32	78.49	-5.66	14.83	-100.6
3.23.21	RAB004R	M	26d	20d	194.53	35.27	6.86	-57.25	175.02	-24.72	16.49	-250.75

Table S10. Raw data for CF versus wildtype (WT) rabbit tracheal explant Ussing measurements reported in Fig. 6, N to S. Baseline short circuit current (Isc) reported in µA/cm^2, baseline resistance (R) reported in Ω/cm^2, baseline potential difference (PD) reported in mV. All other measurements are changes in Isc reported in µA/cm^2.

Ussing Date	Rabbit ID	Sex	Genotype	Age	Days in culture	Isc	R	PD	Amil	FSK	CFTRinh-172	UTP	Bumet
10.27.20	RAB003Q	M	CF	501d	14d	84.88	65.9	5.59	-94.59	-0.9	0	-0.09	1.64
1.25.21	RAB001R	M	CF	430d	14d	59.57	48.68	2.9	-49.09	-1.62	-1.97	16.26	-3.41
1.25.21	RAB001R	M	CF	430d	14d	137.01	55.25	7.57	-120.93	1.14	-2.63	71.64	-5.67
1.28.21	RAB002R	M	WT	433d	14d	126.87	36.33	4.61	-118.44	64.92	-15.61	15.62	-58.76
1.28.21	RAB002R	M	WT	433d	14d	58.35	90.44	5.28	-27.95	18.63	-8.52	2.17	-10.06
3.17.21	RAB003R	M	CF	27d	14d	134.97	27.02	3.65	-100.36	1.33	-5.9	35.88	-21.37
3.17.21	RAB003R	M	CF	27d	14d	199.92	6.2	1.24	-175.36	0	-13.77	46.68	-38.83
3.17.21	RAB004R	M	WT	26d	14d	421.96	84.91	35.83	-134.42	125.21	-37.74	22.42	-324.91
7.21.21	RAB007R		WT	571d	14d	303.21	33.68		-107.58	13.32	-9.21	8.68	-70.38
7.21.21	RAB007R		WT	571d	14d	176.74	139.83	24.71	-84.23	56.76	-20.66	65.12	-70.7
7.28.21	RAB008R	F	CF	578d	14d	219.92	112.21	24.68	-165.86	-0.63	0	34.28	-2.54
7.28.21	RAB008R	F	CF	578d	14d	212.39	155.15	32.95	-177.65	-0.88	0	9.96	-2.01
9.01.21	RAB009R	F	WT	613d	14d	241.96	61.57	14.9	-164.34	86.64	-33.74	20.64	-150.11
9.01.21	RAB009R	F	WT	613d	14d	153.18	40.35	6.18	-130.4	71.37	-15.65	14.58	-98.85
9.15.21	RAB010R	M	CF	662d	14d	107.34	112.89	12.12	-115.74	-2.46	-1.13	21.4	-3.55
9.15.21	RAB010R	M	CF	662d	14d	88.53	149.49	13.23	-95.41	-5.43	0	16.42	-1.4

Table S11. Raw data for post-thaw versus fresh LAE Ussing measurements reported in Fig. 7, M to R. Baseline short circuit current (Isc) reported in μA/cm^2, baseline resistance (R) reported in Ω/cm^2, baseline potential difference (PD) reported in mV. All other measurements are changes in Isc reported in μA/cm^2.

Ussing Date	Donor ID	Tissue	Condition	Days in culture	Isc	R	PD	Amil	FSK	CFTRinh-172	UTP	Bumet
10.11.22	DD042S	LAE	Post-thaw	14d	17.34	48.56	0.84	-1.71	14.53	-12.02	0.58	-2.44
11.07.22	DD046S	LAE	Post-thaw	14d	13.15	31.05	0.41	-5.1	20.22	-18.52	1.97	-3.37
11.07.22	DD046S	LAE	Post-thaw	14d	33.13	38.76	1.28	-6.78	20.99	-11.27	1.4	-4.8
12.06.22	DD050S	LAE	Post-thaw	14d	71.44	12.63	0.9	-45.69	48.82	-27.02	14.1	-5.85
12.06.22	DD050S	LAE	Post-thaw	14d	-7.44	45.29	-0.34	-10.42	10.62	-7.61	3.04	-2.54
1.23.23	DD052S	LAE	Post-thaw	14d	21.22	33.09	0.7	-4.67	18.46	-1.73	1.73	-5.79
1.23.23	DD053S	LAE	Post-thaw	14d	8.3	29.79	0.25	-2.94	5.81	-7	2.58	-1.1
1.23.23	DD053S	LAE	Post-thaw	14d	25.02	35.47	0.89	-5.46	15.54	-19.86	2.7	-4.41
10.12.14	DD062Q	LAE	Fresh	14d	51.13	45.19	2.31	-19.9	16.06	-0.9	4.61	-17.61
2.08.21	DD004R	LAE	Fresh	14d	20.22	33.14	0.67	-4.88	24.1	-2.33	9.78	-1.26
3.04.21	DD007R	LAE	Fresh	14d	58.9	84.8	4.99	-39.12	13.89	-13.36	4.66	-1.35
3.18.21	AA006R	LAE	Fresh	14d	91.47	43.91	4.02	-70.39	36.06	-38.72	4.98	-1.13
3.18.21	AA006R	LAE	Fresh	14d	20.94	46.21	0.97	-26.27	7.9	-11.84	2.68	-0.59
4.30.21	DD015R	LAE	Fresh	14d	40.3	81.43	3.28	-24.81	16.69	-17.18	6.64	-11.44
7.27.22	DD033S	LAE	Fresh	14d	21.26	182.32	3.88	-3.64	8.32	-3.8	1.26	-2.05

Table S12. Raw data for SARS-CoV-2 qRT-PCR reported in Fig. 8, F to G. Ct values were compared to a standard curve to obtain the viral copy number (VCN), which is reported below. Data presented in Fig. 8, F to G are the $Log_2(1+VCN)$.

Gene	Donor ID	LAE Mock	LAE SARS-CoV-2	SAE Mock	SAE SARS-CoV-2
SARS-CoV-2 N1	DD035R	2.36	58741.48	2.52	413525.2
SARS-CoV-2 N1	DD034R	0	549400.8	618.74	291456.8
SARS-CoV-2 N1	AA027R	1.53	331761.9	2.46	241287.2
SARS-CoV-2 N2	DD035R	0	93354.72	1.48	651261.8
SARS-CoV-2 N2	DD034R	0	918839.8	915.89	441323.2
SARS-CoV-2 N2	AA027R	0	539650.1	0	681384.9

Table S13. Raw data for interferon stimulated gene (ISG) qRT-PCR reported in Fig. 8, H to I. *Fold change, reported below,* was *calculated* as 2^(-ΔΔCT). Data presented in Fig. 8, H to I are the $Log_2(1+FC)$.

Gene	Donor ID	LAE Mock	LAE SARS-CoV-2	SAE Mock	SAE SARS-CoV-2
ISG15	DD035R	1.00	7.83	0.61	1.60
ISG15	DD034R	0.45	22.08	9.24	5.15
ISG15	AA027R	2.83	16.62	1.38	2.58
MX1	DD035R	1.00	6.31	0.78	2.54
MX1	DD034R	1.05	17.64	8.10	6.39
MX1	AA027R	1.56	15.57	0.86	14.42

Table S14. Raw data for a-tubulin quantitation reported in Fig. S3, N and Q. *a-tubulin (A-tub) area reported in µm², was normalized to basement membrane (BM) length reported in µm.*

Code	Airway Size	Day	A-Tub Area	BM Length	A-Tub Area / BM Length
DD042S	SAE	0	6533.49	2195.45	2.98
DD042S	SAE	14	4389.98	990.77	4.43
DD042S	LAE	0	18322.5	4346.68	4.21
DD042S	LAE	14	14304.2	5468.06	2.62
DD052S	SAE	0	26746.27	4838.14	5.53
DD052S	SAE	14	31190.5	5912	5.28
DD052S	LAE	0	568.43	784.56	0.72
DD052S	LAE	14	8833.78	3547.42	2.49
DD002S	SAE	0	3483.87	1189.55	2.93
DD002S	SAE	14	3157.51	3825	0.83
DD002S	LAE	0	27568.6	11242.6	2.45
DD002S	LAE	14	20299.2	7927.85	2.56
DD051S	SAE	0	16429	2146.10	7.66
DD051S	SAE	14	6561.95	1719.91	3.81
DD012S	LAE	0	16572	5084.98	3.26
DD012S	LAE	14	3826.54	2184.55	1.75
DD011T	LAE	0	13520.9	5138.83	2.63
DD011T	LAE	14	3005.78	1508.47	1.99

Table S15. Raw data for pig tracheal explant Ussing measurements reported in Fig. S5, A to F. Baseline short circuit current (Isc) reported in µA/cm^2, baseline resistance (R) reported in Ω/cm^2, baseline potential difference (PD) reported in mV. All other measurements are changes in Isc reported in µA/cm^2.

Ussing Date	Donor ID	Days in Culture	Isc	R	PD	Amil	FSK	CFTRinh-172	UTP	Bumet
9.29.21	PIG001R	7d	14.64	117.46	1.72	-12.43	22.77	-0.93	7.94	-12.71
9.29.21	PIG001R	7d	82.45	52.62	4.34	-26.54	43.41	-6.7	15.17	-47.46
9.29.21	PIG002R	7d	83.59	125.62	10.5	-18.73	85.54	-5.65	13.75	-58.14
9.29.21	PIG002R	7d	177.06	62.38	11.05	-28.38	133.21	-14.04	21.4	-95.4
10.06.21	PIG001R	14d	162.28	29.63	4.81	-55.61	37.99	-12.89	5.51	-64.85
10.06.21	PIG001R	14d	64.12	119.5	7.66	-20	58.14	-4.08	13.69	-71.89
10.06.21	PIG002R	14d	71.76	103.29	7.41	-11.65	67.55	3.56	14.17	-67.56
10.06.21	PIG002R	14d	146.05	74.88	10.94	-52.97	94.48	-13.55	13.84	-144.66
10.13.21	PIG001R	21d	53.96	92.72	5	-18.55	26.22	-5.84	11.15	-16.18
13.13.21	PIG002R	21d	18.97	66.07	1.25	-5.51	10.71	-6.88	2.88	-4.4
13.13.21	PIG002R	21d	73.46	52.49	3.86	-15.38	41.98	-12.42	10.69	-24.17
1.27.23	PIG001T	8d	52.61	32.43	1.71	-1.23	4.11	7.47	2.73	1.13
1.27.23	PIG001T	8d	58.46	42.47	2.48	-7.51	20.14	-3.73	5.6	-1.34
1.27.23	PIG002T	7d	64.38	50.38	3.24	-14.48	31.5	-11.95	8.85	-39.06
1.27.23	PIG002T	7d	95.97	43.46	4.17	-27.84	23.66	-15.54	4.72	-33.04
2.02.23	PIG001T	14d	137.66	54.99	7.57	-19.9	22.58	-9.55	6.11	-68.85
2.02.23	PIG001T	14d	74.14	31.2	2.31	-6.88	2.4	16.14	1.79	-4.51
2.02.23	PIG002T	13d	58.66	98.84	5.8	-13.52	59.06	-17.57	11.47	-29.54
2.02.23	PIG002T	13d	27.17	65.13	1.77	-1.99	26.08	-3.88	3.29	-4.89
02.09.23	PIG001T	21d	59.47	50.16	2.98	-13.34	41.28	-21.04	11.76	-4.46
02.09.23	PIG001T	21d	82.93	21.77	1.81	-13.28	16.64	5.56	3.06	-13.58
02.09.23	PIG002T	20d	77.06	47.22	3.64	-23.88	53.46	-12.46	9.16	-1.82
02.09.23	PIG002T	20d	25.93	31.99	0.83	-1.64	16.77	-2.03	0.21	-3.26

Table S16. Raw data for rabbit tracheal explant mucociliary clearance (MCC) measurements reported in Fig. S6A.

Date of measurement	Rabbit ID	Age of rabbit (days)	Sex	Days in culture	Days in culture	MCC (mm/min)
10.7.20	RAB002Q	292	M	het	7d	7.57
10.7.20	RAB002Q	292	M	het	7d	7.11
10.7.20	RAB002Q	292	M	het	7d	10.17
10.7.20	RAB002Q	292	M	het	7d	9.91
11.16.20	RAB005Q	31		het	7d	2.58
1.21.21	RAB002R	433	M	WT	7d	4.33
3.10.21	RAB004R	26	M	WT	7d	0
4.15.21	RAB005R	428	M	het	7d	11.05
8.25.21	RAB009R	613	F	het	7d	6.43
8.25.21	RAB009R	613	F	het	7d	3.34
10.27.20	RAB004Q	586	M	het	7d	10.46
10.14.20	RAB002Q	292	M	het	14d	7.9
10.14.20	RAB002Q	292	M	het	14d	6.65
11.23.20	RAB005Q	31		het	14d	3.27
1.28.21	RAB002R	433	M	WT	14d	6.37
1.28.21	RAB002R	433	M	WT	14d	9.42
3.17.21	RAB004R	26	M	WT	14d	0.97
4.22.21	RAB005R	428	M	het	14d	0
4.22.21	RAB005R	428	M	het	14d	10.97
7.21.21	RAB007R	571	F	het	14d	3.25
7.21.21	RAB007R	571	F	het	14d	6.49
7.21.21	RAB007R	571	F	het	14d	3.17
7.21.21	RAB007R	571	F	het	14d	4.87
9.1.21	RAB009R	613	F	het	14d	14
9.1.21	RAB009R	613	F	het	14d	9
10.21.20	RAB002Q	292	M	het	21d	13.4
10.21.20	RAB002Q	292	M	het	21d	13.32
10.21.20	RAB002Q	292	M	het	21d	7.66
10.21.20	RAB002Q	292	M	het	21d	13.51
2.4.21	RAB002R	433	M	WT	21d	9.43
2.4.21	RAB002R	433	M	WT	21d	5.48
3.23.21	RAB004R	26	M	WT	21d	5.52
4.29.21	RAB005R	428	M	het	21d	5.94
4.29.21	RAB005R	428	M	het	21d	14.02

Table S17. Raw data for surface area of rabbit tracheal explants over time, reported in fig. S6B. Data in fig. S6B and displayed below has been normalized to the day 0 surface area and expressed as a percentage.

**Explant**	**Day 0 (%)**	**Day 7 (%)**	**Day 15-16 (%)**
**Replicate 1**	100	65.46	56.5
**Replicate 2**	100	67.76	56.2
**Replicate 3**	100	71.02	58.6
**Replicate 4**	100	66.44	51.1
**Replicate 5**	100	62.25	50.2
**Replicate 6**	100	67.5	50.5

Table S18. Raw data for rabbit tracheal explants made from cryopreserved tissue. Ussing measurements reported in fig. S6, F to K. Baseline short circuit current (Isc) reported in μA/cm^2, baseline resistance (R) reported in Ω/cm^2, baseline potential difference (PD) reported in mV. All other measurements are changes in Isc reported in μA/cm^2.

Ussing Date	Genotype	Rabbit ID	Sex	Age of rabbit (days)	Days in culture	Isc	R	PD	Amil	FSK	CFTRinh-172	UTP	Bumet
8.9.22	WT	4973	F	86	7d	213.07	105.8	22.54	-118.01	31.88	-26.23	13.96	-90.26
8.9.22	WT	4973	F	86	7d	193.95	59.1	11.46	-99.29	21.54	-13.43	12.6	-93.96
12.13.22	WT		M	139	7d	50.26	34.6	1.74	-23.25	12.85	-4.82	2.19	-28.03
12.13.22	WT		M	139	7d	350.11	62.11	21.75	-234.42	80.45	-56.53	14.57	-137.66
12.13.22	WT		M	139	7d	349.66	67.72	23.68	-231.77	101.36	-66.87	11.17	-160.3
1.12.23	WT	7248	M	139	7d	167.56	85.15	14.27	-88.95	22.43	-17.37	10.78	-89.4
1.12.23	WT	7248	M	139	7d	160.11	154.79	24.78	-93.39	48.41	-26.63	5.4	-70.79
1.17.23	WT	4940	F	350	7d	137.04	134.11	18.38	-43.18	36.59	-20.77	3.55	-93.45
1.17.23	WT	4940	F	350	7d	228.2	92.79	21.17	-88.77	65.24	-38.16	4.83	-149.03
12.13.22	CF	7223	M	137	7d	41.83	24.09	1.01	-19.41	0.14	0	4.49	-2.14
12.13.22	CF	7223	M	137	7d	25.16	28.76	0.72	-21.06	0	-1.06	1.85	-3.39
12.13.22	CF	7223	M	137	7d	109.65	94.24	10.33	-95.67	-0.26	0	2.58	-5.52
01.12.23	CF	7248	M	139	7d	205.96	181.01	37.28	-192.18	0	0	3.71	-8.03
01.12.23	CF	7248	M	139	7d	166.4	75.28	12.30	-153.66	0	0	3.67	-6.79
01.17.23	CF	7222	M	155	7d	43.31	144.49	6.26	-34.76	0	-1.34	1.06	-2.29

Table S19. Raw data for SARS-CoV-2 qRT-PCR reported in fig. S7, A to B. Ct values were compared to a standard curve to obtain the viral copy number (VCN), which is reported below. Data presented in fig. S7, A to B are the Log2(1+VCN). For the calculation of ΔΔCt values, undetectable values were assigned Ct=40, corresponding to the maximum number of amplification cycles.

Gene	Donor ID	Mock (d1-2)	SARS-CoV-2 (d1-2)	Mock (d16-24)	SARS-CoV-2 (d16-24)
SARS-CoV-2 N1	DD029U	0.52	42827.32	1.97	74324.21
SARS-CoV-2 N1	DD028U	0.52	3297.62	4.66	119493.1
SARS-CoV-2 N1	DD024U	0.52	495080.10	0.52	
SARS-CoV-2 N2	DD029U	33.97	2145153.79	67.92	1869001.98
SARS-CoV-2 N2	DD028U	67.92	1414.02	67.92	1519787.68
SARS-CoV-2 N2	DD024U	33.99	32246362.75	67.92	

Table S20. Raw data for interferon stimulated gene (ISG) qRT-PCR reported in fig. S7, C to D. *Fold change, reported below,* was *calculated* as 2^(-ΔΔCT). Data presented in fig. S7, C to D are the $Log_2(1+FC)$.

Gene	Donor ID	Mock (d1-2)	SARS-CoV-2 (d1-2)	Mock (d16-24)	SARS-CoV-2 (d16-24)
ISG15	DD029U	1.64	6.19	1.97	5.88
ISG15	DD028U	1.33	1.32	0.93	7.07
ISG15	DD024U	1.00	4.44	0.95	
MX1	DD029U	2.43	7.44	1.28	4.61
MX1	DD028U	1.38	1.66	1.11	5.73
MX1	DD024U	1.00	33.23	1.382	
